# Supplementary material for: A genome-wide association analysis identifies 16 novel susceptibility loci for carpal tunnel syndrome
Source: Nat Commun. 2019 Mar 4;10:1030. doi: 10.1038/s41467-019-08993-6 (PMC6399342; doi:10.1038/s41467-019-08993-6)
Supplement: Supplementary file 1 — Supplementary Information [file 41467_2019_8993_MOESM1_ESM.pdf]

Supplementary Information

Supplement to:

Wiberg et al. A genome-wide association analysis identifies 16 novel susceptibility loci for Carpal Tunnel Syndrome.

Table of Contents

Supplementary Table 1. Diagnostic codes used for CTS case definition

Supplementary Table 2. Gene-based association analysis in MAGMA

Supplementary Table 3. Gene-based enrichment analysis in FUMA

Supplementary Table 4. Gene-based enrichment analysis in XGR

Supplementary Table 5. Summary-based Mendelian Randomisation (SMR) using eQTL data from GTEx v6 transformed fibroblasts

Supplementary Table 6. Partitioned CTS heritability across different cell and tissue types

Supplementary Table 7. Genetic correlation between CTS and other phenotypes

Supplementary Table 8. Gene expression of *COL11A1* and *EFEMP1* in tibial nerve and transformed fibroblasts

Supplementary Table 9. eQTLs for functional SNPs at 2p16.1 (*EFEMP1* locus)

Supplementary Figure 1. Overview of Quality Control (QC)

Supplementary Figure 2. Heat map of gene expression in 53 tissue types

Supplementary Figure 3. Gene-based association analysis Manhattan plot

Supplementary Figure 4. Gene-property analysis in MAGMA

Supplementary Figure 5. Partitioned CTS heritability across genomic functional categories

Supplementary Figure 6. Hierarchical clustering of samples in RNA-Seq

Supplementary Figure 7. Regional plots for all GWAS-associated loci

Supplementary Discussion

Supplementary References

Supplementary Table 1. Diagnostic codes used for CTS case definition. The number of individuals with each of the diagnostic codes are shown below. Following sample QC, a total of 12,312 individuals had at least one of the diagnostic codes for CTS.

Carpal Tunnel Syndrome

Source of Data	UK Biobank Data Field	Code	Description	N
Primary ICD-10	41202	G560	Carpal Tunnel Syndrome	9139
Secondary ICD-10	41204	G560	As above	648
Primary OPCS	41200	A651	Carpal Tunnel Release	9071
		A692	Revision of Carpal Tunnel Release	97
Secondary OPCS	41210	A651	Carpal Tunnel Release	452
		A692	Revision of Carpal Tunnel Release	7
Non-cancer illness (self-report)	20002	1541	Carpal Tunnel Syndrome	965
Operation (self-report)	20004	1501	Carpal Tunnel Surgery	4584
**Total (excluding overlaps)**				**12312**

Supplementary Table 2. Gene-based association analysis in MAGMA. Seventeen genes met the threshold for genome-wide significance ($p<2.68\times10^{-6}$) in this analysis.

Gene[a]	Chromosome	P-value
LOXL4	10	9.47×10^{-9}
R3HCC1L	10	7.08×10^{-8}
COL11A1	1	1.07×10^{-7}
SMAD6	15	1.59×10^{-7}
PTPN9	15	1.66×10^{-7}
HAS1	19	1.87×10^{-7}
ZBTB38	3	2.13×10^{-7}
AFF1	4	3.80×10^{-7}
ADAMTS17	15	4.88×10^{-7}
PBX3	9	4.90×10^{-7}
KIAA1715	2	5.83×10^{-7}
XRCC6	22	7.30×10^{-7}
AOC1	7	1.10×10^{-6}
CCDC134	22	1.69×10^{-6}
AEBP1	7	2.32×10^{-6}
EFEMP1	2	2.40×10^{-6}
SIN3A	15	2.55×10^{-6}

[a]Genes highlighted in bold were also prioritised by positional mapping in FUMA.

Supplementary Table 3. Gene-based enrichment analysis in FUMA. This analysis was performed using the GENE2FUNC tool in FUMA, using the 25 positionally mapped genes in FUMA and the 17 genes from the MAGMA gene-based analysis. N = number of genes in the ontology; n = number of overlapped genes.

25 genes from FUMA positional mapping

GO Cellular components

GeneSet	N	n	P-value	Adjusted P-value	Genes
GO_EXTRACELLULAR_MATRIX	424	7	4.67×10^{-11}	2.71×10^{-8}	*COL11A1, TGFB3, ADAMTS17, ADAMTS10, LTBP1, EFEMP1, AEBP1*
GO_PROTEINACEOUS_EXTRACELLULAR_MATRIX	355	5	3.50×10^{-8}	6.77×10^{-6}	*COL11A1, ADAMTS17, ADAMTS10, LTBP1, EFEMP1*
GO_EXTRACELLULAR_SPACE	1367	5	8.30×10^{-5}	4.81×10^{-3}	*TGFB3, EFEMP1, MUC13, AEBP1, AOC1*
GO_CYTOSKELETON	1965	5	5.95×10^{-4}	2.30×10^{-2}	*TTLL5, MYO1F, KALRN, MYL7, GCK*

GWAS Catalog Reported Genes

Gene Set	N	n	P-value	Adjusted P-value	Genes
Waist circumference adjusted for body mass index	126	5	7.01×10^{-11}	8.57×10^{-8}	*ADAMTS17, MYO1F, ADAMTS10, LTBP1, EFEMP1*
Height	522	7	2.42×10^{-10}	1.48×10^{-7}	*TTLL5, ADAMTS17, MYO1F, ADAMTS10, LTBP1, EFEMP1, TMEM176A*

17 genes from MAGMA gene-based analysis

GO Cellular components

Gene set	N	n	P-value	Adjusted P-value	Genes
GO_EXTRACELLULAR_SPACE	1367	5	2.93×10^{-5}	1.55×10^{-3}	*LOXL4, EFEMP1, ZBTB38, AEBP1, AOC1*

GWAS Catalog Reported Genes

Gene set	N	n	P-value	Adjusted P-value	Genes
Height	522	5	1.12×10^{-7}	4.54×10^{-5}	*SIN3A, PTPN9, ADAMTS17, EFEMP1, ZBTB38*

Supplementary Table 4. Gene-based enrichment analysis in XGR. We used the XGR Gene-based Enrichment Analysis function (selecting 'canonical pathways') to classify the 25 genes mapped by FUMA into different functional categories. The Z-scores, p-values, FDR, and the overlapped genes for each of the ontologies are shown.

Term Name	Z-Score	P-value	FDR	Number of overlapped genes	Genes
Ensemble of genes encoding core extracellular matrix including ECM glycoproteins, collagens and proteoglycans	3.24	0.0016	0.0032	4	*AEBP1, COL11A1, EFEMP1, LTBP1*
Ensemble of genes encoding extracellular matrix and extracellular matrix-associated proteins	2.79	0.0016	0.0032	8	*ADAMTS10, ADAMTS17, AEBP1, COL11A1, EFEMP1, LTBP1, MUC13, TGFB3*
Genes encoding structural ECM glycoproteins	2.89	0.0035	0.0047	3	*AEBP1, EFEMP1, LTBP1*
Ensemble of genes encoding ECM-associated proteins including ECM-affiliated proteins, ECM regulators and secreted factors	0.962	0.1	0.1	4	*ADAMTS10, ADAMTS17, MUC13, TGFB3*

Supplementary Table 5. Summary-based Mendelian Randomisation (SMR) using eQTL data from GTEx v6 transformed fibroblasts. Columns are: Probe ID, probe chromosome, gene name, SNP name, effect allele, non-effect allele, frequency of the effect allele, GWAS p-value, eQTL p-value, SMR p-value and HEIDI p-value (see Methods). The two probes (genes) that met the Bonferroni-corrected significance threshold are shown.

Probe ID	Chr	Gene	Top SNP	A1	A2	Freq	P_{GWAS}	P_{eQTL}	P_{SMR}	P_{HEIDI}
ENSG00000049323.11	2	*LTBP1*	rs7571401	C	G	0.555473	8.10×10^{-7}	2.30×10^{-47}	3.03×10^{-6}	0.530
ENSG00000140400.10	15	*MAN2C1*	rs10220738	T	A	0.747759	2.20×10^{-6}	6.48×10^{-63}	5.25×10^{-6}	0.968

Supplementary Table 6. Partitioned CTS heritability across different cell and tissue types. This analysis was implemented using LDSC-SEG across 205 different tissue and cell types from GTEx and Franke Lab gene expression datasets. The cell and tissue types with coefficient p-value<0.05 are shown (with regression coefficients and standard errors). Additionally, the cell and tissue types have been classified into nine distinct tissue categories, as per Finucane et al.

Name	Coefficient	Coefficient SE	Coefficient P-value	Tissue category
A11.329.629.Osteoblasts	2.72×10^{-9}	8.32×10^{-10}	0.000544043	Musculoskletal/Connective
A11.620.520.Myocytes..Smooth.Muscle	2.14×10^{-9}	7.76×10^{-10}	0.002915213	Musculoskletal/Connective
A11.872.190.260.Embryoid.Bodies	2.56×10^{-9}	9.31×10^{-10}	0.002963495	Other
A10.165.114.830.750.Subcutaneous.Fat	1.56×10^{-9}	6.24×10^{-10}	0.006340782	Adipose
A10.165.450.300.Cicatrix	1.55×10^{-9}	6.27×10^{-10}	0.006780791	Musculoskletal/Connective
Artery_Tibial	1.42×10^{-9}	6.11×10^{-10}	0.010118058	Cardiovascular
A11.329.171.Chondrocytes	1.94×10^{-9}	8.39×10^{-10}	0.010502383	Musculoskletal/Connective
Artery_Aorta	1.45×10^{-9}	6.38×10^{-10}	0.011613989	Cardiovascular
A02.165.Cartilage	1.80×10^{-9}	8.19×10^{-10}	0.013885044	Musculoskletal/Connective
A05.360.319.679.690.Myometrium	1.52×10^{-9}	6.93×10^{-10}	0.014279534	Musculoskletal/Connective
A08.186.211.653.Mesencephalon	1.45×10^{-9}	6.88×10^{-10}	0.017696367	CNS
A10.690.467.Muscle..Smooth	1.46×10^{-9}	7.48×10^{-10}	0.025717943	Musculoskletal/Connective
A11.872.580.Mesenchymal.Stem.Cells	1.37×10^{-9}	7.37×10^{-10}	0.031147321	Other
A11.118.637.555.567.562.B.Lymphocytes	1.52×10^{-9}	8.26×10^{-10}	0.032722413	Blood/Immune
A10.165.450.300.425.Keloid	1.10×10^{-9}	6.08×10^{-10}	0.034930974	Musculoskletal/Connective
Adipose_Subcutaneous	1.14×10^{-9}	6.46×10^{-10}	0.039305727	Adipose
A10.165.114.830.500.750.Subcutaneous.Fat..Abdominal	1.12×10^{-9}	6.36×10^{-10}	0.039818441	Adipose
A10.615.789.Serous.Membrane	1.42×10^{-9}	8.26×10^{-10}	0.042352467	Other
A03.556.500.760.464.Parotid.Gland	1.21×10^{-9}	7.33×10^{-10}	0.049218223	Digestive

Supplementary Table 7. Genetic correlation between CTS and other phenotypes. The analysis was performed using LD score (LDSC) regression, implemented in LD Hub. The traits are shown along with the PMID of the study from which the LDSC data were derived, the trait category, and the correlation coefficient (r_g). Traits are ranked by p-value, and the top 14 traits meet a Bonferroni-corrected significant threshold of p<0.0014.

Trait 1	Trait 2	PMID	Category	rg	se	z	p	h2_obs	h2_obs_se	h2_int	h2_int_se	gcov_int	gcov_int_se
**CTS**	Body mass index	20935630	anthropometric	0.3464	0.0351	9.8663	5.83E-23	0.1906	0.0095	1.0089	0.0106	-0.0078	0.0061
**CTS**	Overweight	23563607	anthropometric	0.3613	0.0376	9.6148	6.92E-22	0.1099	0.0065	1.0235	0.0096	-0.0074	0.0059
**CTS**	Obesity class 1	23563607	anthropometric	0.3268	0.0382	8.5645	1.09E-17	0.2179	0.0118	1.0168	0.0111	-0.0003	0.0068
**CTS**	Waist-to-hip ratio	25673412	anthropometric	0.2465	0.035	7.0465	1.83E-12	0.1135	0.007	0.9197	0.0092	-0.0051	0.0055
**CTS**	Obesity class 2	23563607	anthropometric	0.3176	0.0482	6.5887	4.44E-11	0.1856	0.0127	1.0004	0.01	0.0013	0.0067
**CTS**	Height_2010	20881960	anthropometric	-0.2168	0.0368	-5.8985	3.67E-09	0.2859	0.0167	1.0176	0.019	-0.0047	0.0071
**CTS**	Waist circumference	25673412	anthropometric	0.208	0.037	5.6262	1.84E-08	0.1215	0.0052	0.8444	0.0079	-0.0016	0.0054
**CTS**	Extreme bmi	23563607	anthropometric	0.2726	0.0548	4.9694	6.72E-07	0.7009	0.0541	1.0271	0.0113	0.0006	0.0075
**CTS**	Extreme height	23563607	anthropometric	-0.2235	0.0472	-4.7405	2.13E-06	1.2395	0.1072	1.034	0.0177	-0.0008	0.0091
**CTS**	Obesity class 3	23563607	anthropometric	0.2928	0.0681	4.3022	1.69E-05	0.1213	0.0144	0.9802	0.0093	0.0031	0.0065
**CTS**	HbA1C	20858683	glycemic	0.2824	0.0711	3.9739	7.07E-05	0.0655	0.0121	0.9988	0.0073	-0.0036	0.0049
**CTS**	Lumbar spine bone mineral density	22504420	bone	0.1826	0.0483	3.7848	0.0002	0.2669	0.0249	1.0155	0.0085	-0.0083	0.0053
**CTS**	Childhood obesity	22484627	anthropometric	0.2054	0.0575	3.5712	0.0004	0.418	0.0451	0.9251	0.0078	0.0069	0.005
**CTS**	Hip circumference	25673412	anthropometric	0.1235	0.037	3.3331	0.0009	0.1277	0.0059	0.86	0.0081	-0.0004	0.0054
**CTS**	Lumbar Spine bone mineral density	26367794	bone	0.156	0.0498	3.1304	0.0017	0.1278	0.0152	0.9806	0.0082	-0.0004	0.0046
**CTS**	Child birth weight	23202124	anthropometric	-0.2127	0.0694	-3.0639	0.0022	0.1114	0.0185	1.005	0.0061	0.003	0.0048
**CTS**	Fasting glucose main effect	22581228	glycemic	0.1493	0.0522	2.8595	0.0042	0.0968	0.0191	1	0.0099	-0.0016	0.0047
**CTS**	Difference in height between adolescence and adulthood; age 14	23449627	anthropometric	-0.2452	0.0943	-2.5995	0.0093	0.441	0.1077	0.9823	0.0067	0.0053	0.0053
**CTS**	Rheumatoid Arthritis	24390342	autoimmune	0.1132	0.0439	2.582	0.0098	0.1598	0.0293	1.0238	0.017	0.0039	0.0052
**CTS**	Type 2 Diabetes	22885922	glycemic	0.1407	0.0556	2.53	0.0114	0.0887	0.0094	1.0072	0.0072	0.0016	0.0051
**CTS**	Difference in height between childhood and adulthood; age 8	23449627	anthropometric	-0.169	0.0709	-2.3835	0.0172	0.3417	0.0515	0.9704	0.0077	-0.0057	0.0056
**CTS**	Birth weight	27680694	anthropometric	-0.0997	0.0422	-2.3632	0.0181	0.103	0.0068	1.0408	0.0101	-0.0035	0.0053
**CTS**	Child birth length	25281659	anthropometric	-0.1402	0.0727	-1.9288	0.0538	0.1696	0.0231	0.9926	0.0071	-0.0008	0.0059

**CTS**	Serum Urate overweight	25811787	uric_acid	-0.0988	0.0539	-1.8344	0.0666	0.5161	0.2982	0.9708	0.0268	0.0033	0.0051
**CTS**	Femoral Neck bone mineral density	26367794	bone	0.0966	0.0536	1.8024	0.0715	0.1241	0.0147	0.9772	0.0082	0.0001	0.0047
**CTS**	Fasting insulin main effect	22581228	glycemic	0.1117	0.0669	1.6691	0.0951	0.0707	0.0103	1.015	0.0073	0.0002	0.0053
**CTS**	Height; Females at age 10 and males at age 12	23449627	anthropometric	-0.0849	0.0561	-1.513	0.1303	0.429	0.0482	0.9529	0.008	-0.0024	0.0056
**CTS**	Femoral neck bone mineral density	22504420	bone	0.0624	0.0454	1.3744	0.1693	0.3067	0.0258	0.9784	0.0085	0.0063	0.0049
**CTS**	Fasting proinsulin	20081858	glycemic	0.1263	0.0938	1.3474	0.1779	0.1808	0.0894	0.9827	0.0097	-0.0081	0.0052
**CTS**	Extreme waist-to-hip ratio	23563607	anthropometric	0.1121	0.089	1.2601	0.2076	0.3617	0.0585	0.9769	0.0086	0.0047	0.0069
**CTS**	Forearm Bone mineral density	26367794	bone	0.171	0.1389	1.2306	0.2185	0.0874	0.0446	1.0118	0.007	-0.0007	0.0051
**CTS**	Infant head circumference	22504419	anthropometric	-0.0945	0.0799	-1.1829	0.2368	0.2282	0.0443	0.9908	0.0068	0.0003	0.0052
**CTS**	HOMA-IR	20081858	glycemic	0.0869	0.0807	1.0769	0.2815	0.0694	0.0127	1.0025	0.0066	0.004	0.0053
**CTS**	Sitting height ratio	25865494	anthropometric	-0.064	0.0609	-1.0507	0.2934	0.2203	0.0283	0.9802	0.0076	0.0072	0.0054
**CTS**	2hr glucose adjusted for BMI	20081857	glycemic	-0.0732	0.1064	-0.688	0.4915	0.103	0.0345	0.9932	0.0071	0.0016	0.0054
**CTS**	HOMA-B	20081858	glycemic	0.0051	0.0704	0.0718	0.9428	0.0887	0.014	0.9904	0.0066	0.0031	0.0054

Supplementary Table 8. Gene expression of *COL11A1* and *EFEMP1* in tibial nerve and transformed fibroblasts

Gene	Expression in tibial nerve[a]	Rank (tibial nerve)[b]	Expression in transformed fibroblasts[a]	Rank (transformed fibroblasts)[b]
COL11A1	8.727	3	32.24	1
EFEMP1	129.05	13	451.2	4

[a]Exon expression for each candidate gene, measured in transcripts per million (TPM). Data Source: GTEx Analysis Release v7 (dbGaP Accession phs000424.v7.p2).
[b]Rank of exon expression quantity (in TPM) for tibial nerve and transformed fibroblasts, out of 53 tissue types represented in GTEx v7.

Supplementary Table 9. eQTLs for functional SNPs at 2p16.1 (*EFEMP1* locus)

SNP[a]	Gene	p-value	Normalised Effect Size[b]	Tissue	r^2 with rs3791679[c]
**rs3791679**	*EFEMP1*	2.0×10^{-13}	0.32	Thyroid	1
	EFEMP1	1.5×10^{-6}	0.25	Skin – Not Sun Exposed (Suprapubic)	
	CLHC1	4.4×10^{-5}	0.29	Whole Blood	
	EFEMP1	6.1×10^{-5}	0.19	Skin – Sun Exposed (Lower leg)	
**rs6752931**	*EFEMP1*	1.1×10^{-16}	0.34	Thyroid	0.76
**rs4146922**	*EFEMP1*	7.2×10^{-16}	0.35	Thyroid	0.82
	CLHC1	1.1×10^{-5}	0.32	Whole Blood	
	RPS27A	2.2×10^{-5}	-0.21	Colon - Sigmoid	
**rs58680090**	*EFEMP1*	1.3×10^{-16}	0.36	Thyroid	0.86
	CLHC1	2.6×10^{-5}	0.31	Whole Blood	
	RPS27A	3.7×10-5	-0.21	Colon - Sigmoid	
**rs1344732**	*EFEMP1*	3.1×10^{-13}	0.28	Thyroid	0.67
**rs7563032**	*EFEMP1*	1.1×10^{-15}	0.35	Thyroid	0.83
	CLHC1	2.2×10^{-5}	0.31	Whole Blood	
	RPS27A	3.3×10^{-5}	-0.21	Colon - Sigmoid	
**rs6739641**	*EFEMP1*	4.0×10^{-13}	0.28	Thyroid	0.67
	CLHC1	4.5×10^{-5}	0.26	Whole Blood	

Notes: eQTLs were obtained from GTEx v7.
[a]The lead SNP at this locus is rs3791679. The remaining SNPs were chosen on the basis of being both genome-wide significant in the CTS GWAS and having likely functional consequences (i.e. CADD score > 12.37 or RegulomeDB score of 2b or more).
[b]The normalised effect size of the eQTLs is defined as the slope of the linear regression, and is computed as the effect of the alternative allele relative to the reference allele in the human genome reference GRCh37/hg19.
[c]The LD relationship between rs3791679 and the other SNPs was calculated using 1000 Genomes GBR data.

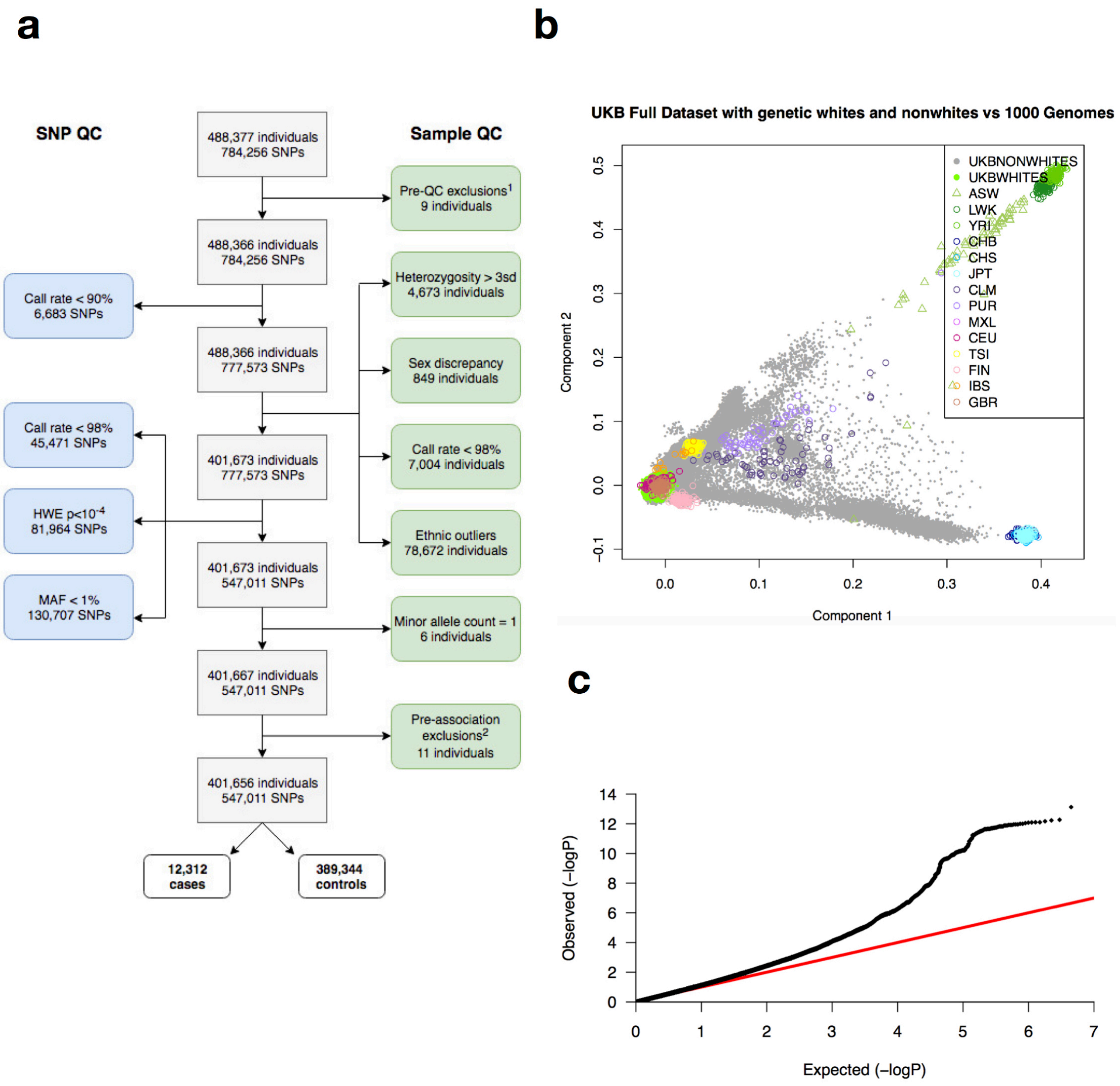

Supplementary Figure 1. Overview of Quality Control (QC). a Flowchart summarising QC protocol. Excluded SNPs are in blue panels on the left and excluded individuals are in green panels on the right. [1]Pre-QC exclusions: 3 individuals with invalid IDs and sex, and 8 individuals who have withdrawn from UK Biobank were excluded prior to QC. [2]Pre-association exclusions: 11 individuals who were not present in UK Biobank's sample file accompanying the BGEN files were excluded prior to association. **b** Principal Component Analysis (PCA) for demonstration of ethnicity of UK Biobank individuals. The UK Biobank cohort was merged with publicly available data from the 1000 Genomes Project and PCA was performed using flashpca. Individuals identified by UK Biobank as having white British ancestry are coloured in lime green, and the remaining UK Biobank individuals are in grey. In this graph of principal component 1 vs principal component 2, a near-perfect overlap can be seen between the UK Biobank "white British" individuals and both GBR (British in England and Scotland - light brown) and CEU (Utah residents with Northern and Western European ancestry - magenta) individuals from the 1000 Genomes Project. **c** QQ (quantile-quantile) plot of associated SNPs. The λ_{GC} demonstrated some inflation (1.15), but the LD score regression (LDSC) intercept of 1.015, with an attenuation ratio of 0.073 indicated that the inflation was largely due to polygenicity and the large sample size.

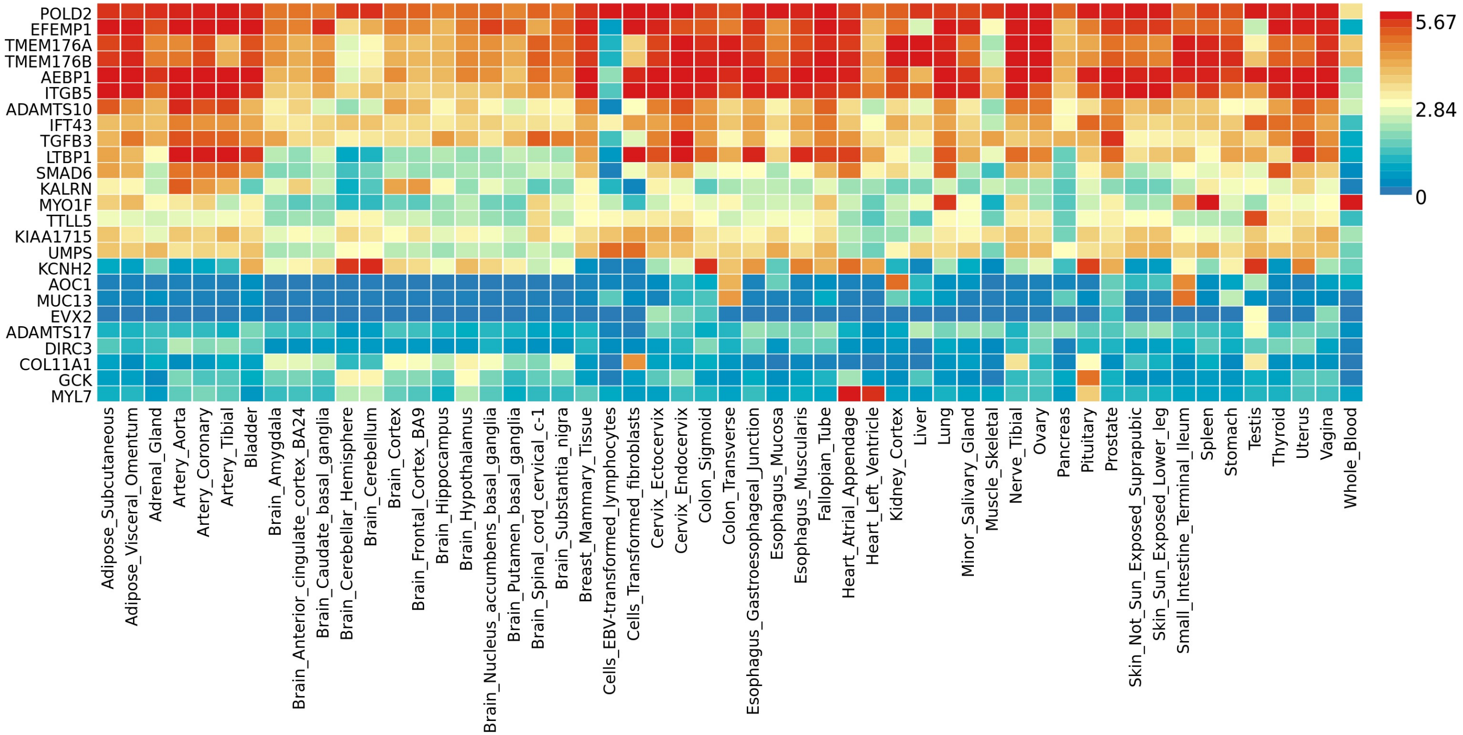

Supplementary Figure 2. Heat map of gene expression across 53 tissue types. This analysis was implemented in FUMA, and demonstrates the average expression values of the 25 genes positionally mapped by FUMA, across 53 tissue types in GTEx v7. This is an averaged expression value per tissue type per gene following winsorization at 50 and log 2 transformation with pseudocount 1. The expression value is in Transcripts per Million, and genes have been organised by hierarchical clustering.

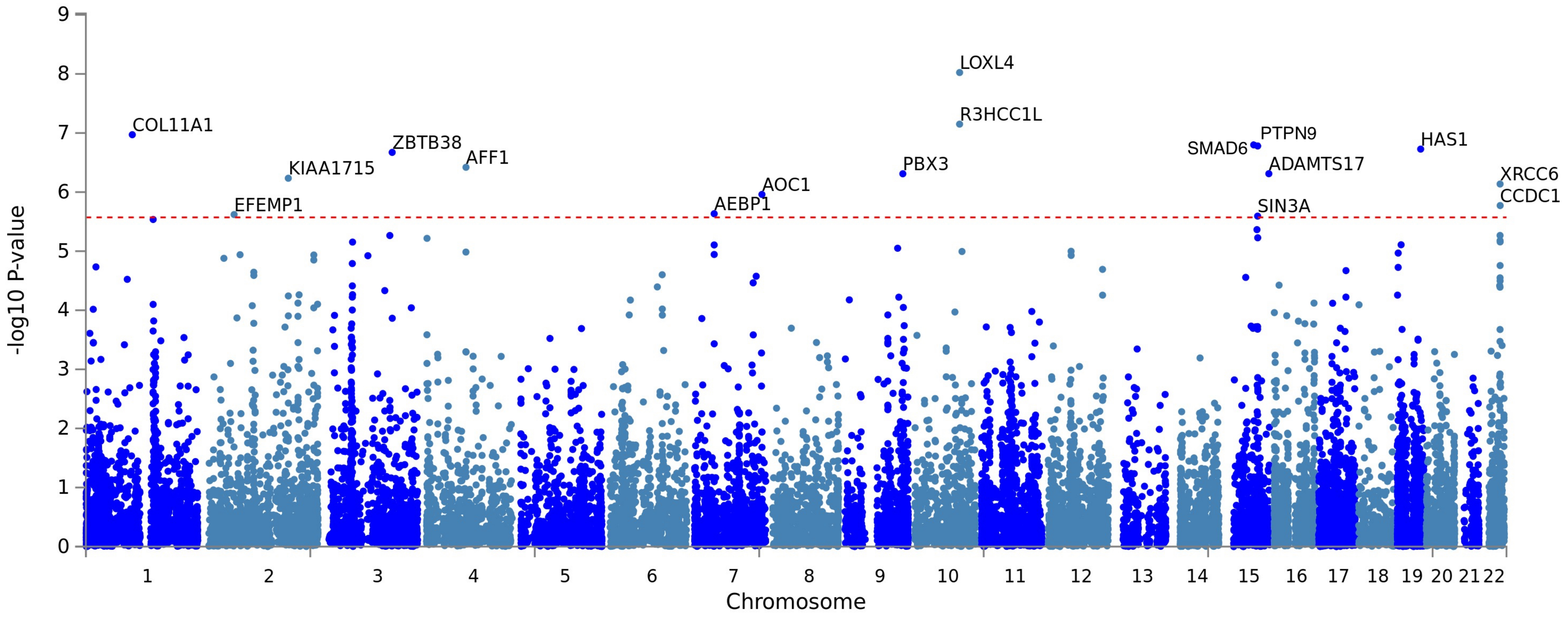

Supplementary Figure 3. Gene-based association analysis Manhattan plot. Association results for CTS in MAGMA gene-based association analysis. The dotted red line indicates the threshold for genome-wide significance ($p<2.68\times10^{-6}$). Seventeen genes (labelled) reach genome-wide significance in this analysis. Source data are provided as a Source Data file.

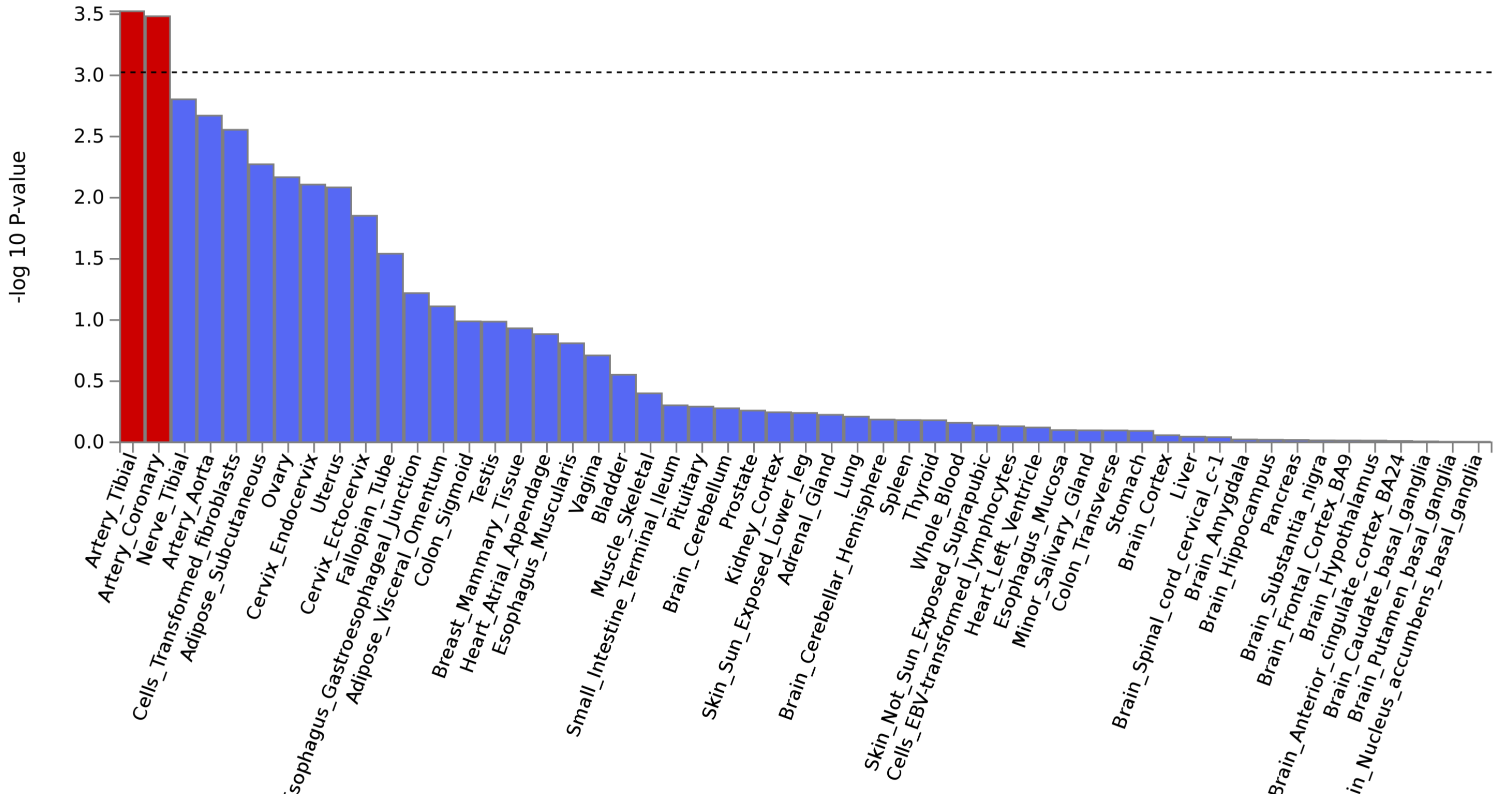

Supplementary Figure 4. Gene-property analysis in MAGMA. MAGMA Tissue Expression Analysis of CTS GWAS-summary data, implemented in FUMA. This tests the relationship between highly expressed genes in a specific tissue and the genetic associations from the GWAS. Gene-property analysis is performed using average expression of genes per tissue type as a gene covariate. Gene expression values are log2 transformed average RPKM (Read Per Kilobase Per Million) per tissue type after winsorization at 50, and are based on GTEx v6 RNA-Seq data across 53 specific tissue types. The dotted line indicates the Bonferroni-corrected α level, and the tissues that meet this significance threshold are highlighted in red. Source data are provided as a Source Data file.

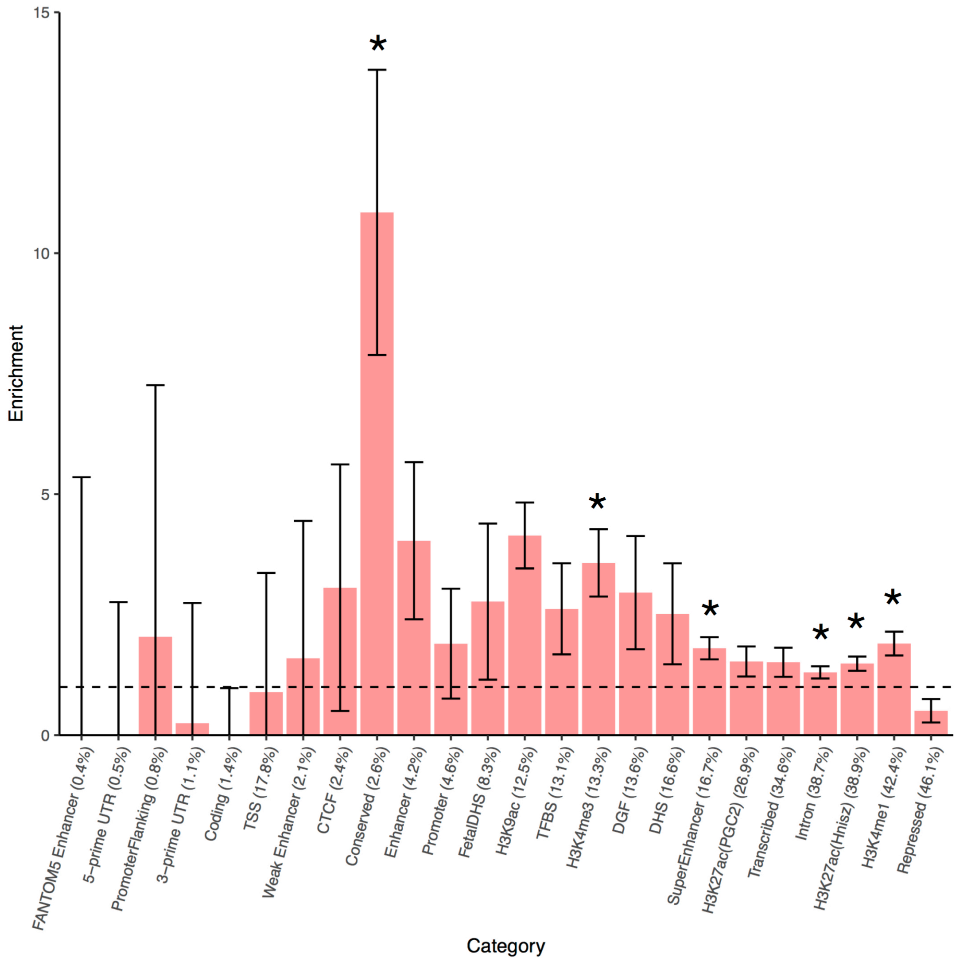

Supplementary Figure 5. Partitioned CTS heritability across genomic functional categories. Enrichment estimates across the 24 main functional annotation categories for CTS heritability. The enrichment statistic is the proportion of heritability found in each functional group divided by the proportion of SNPs in each group. The dashed line indicates an enrichment proportion of 1 (i.e. no enrichment). Error bars represent jackknife standard errors around the enrichment estimates, and an asterisk indicates significance at $p<0.05$ after Bonferroni correction for the 24 annotations tested. Statistical significance is indicated with an asterisk. Source data are provided as a Source Data file.

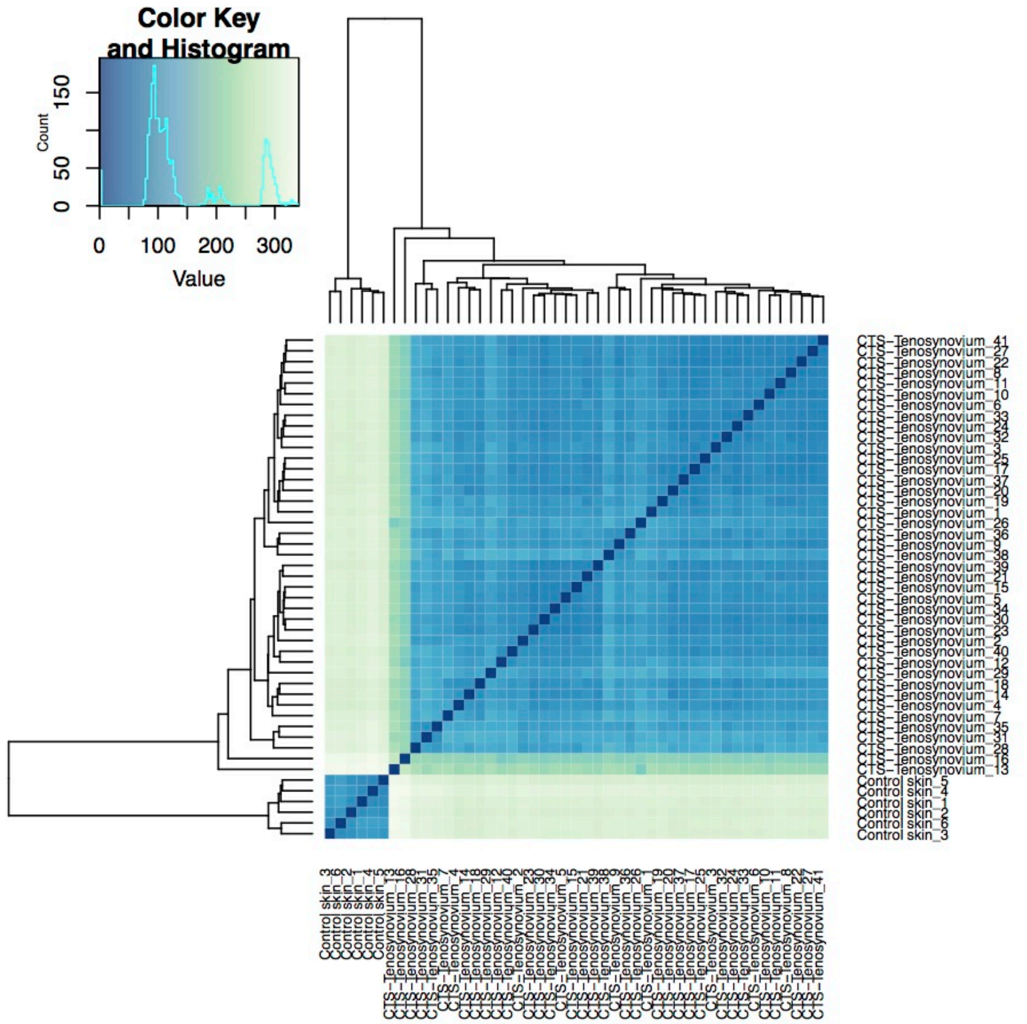

Supplementary Figure 6. Hierarchical clustering of samples. Hierarchical clustering of 41 tenosynovium samples from CTS patients and 6 index finger skin samples from healthy individuals, based on regularised log2 counts of Ensembl genes. Euclidean distances were calculated and clustering was carried out using the Ward criterion.

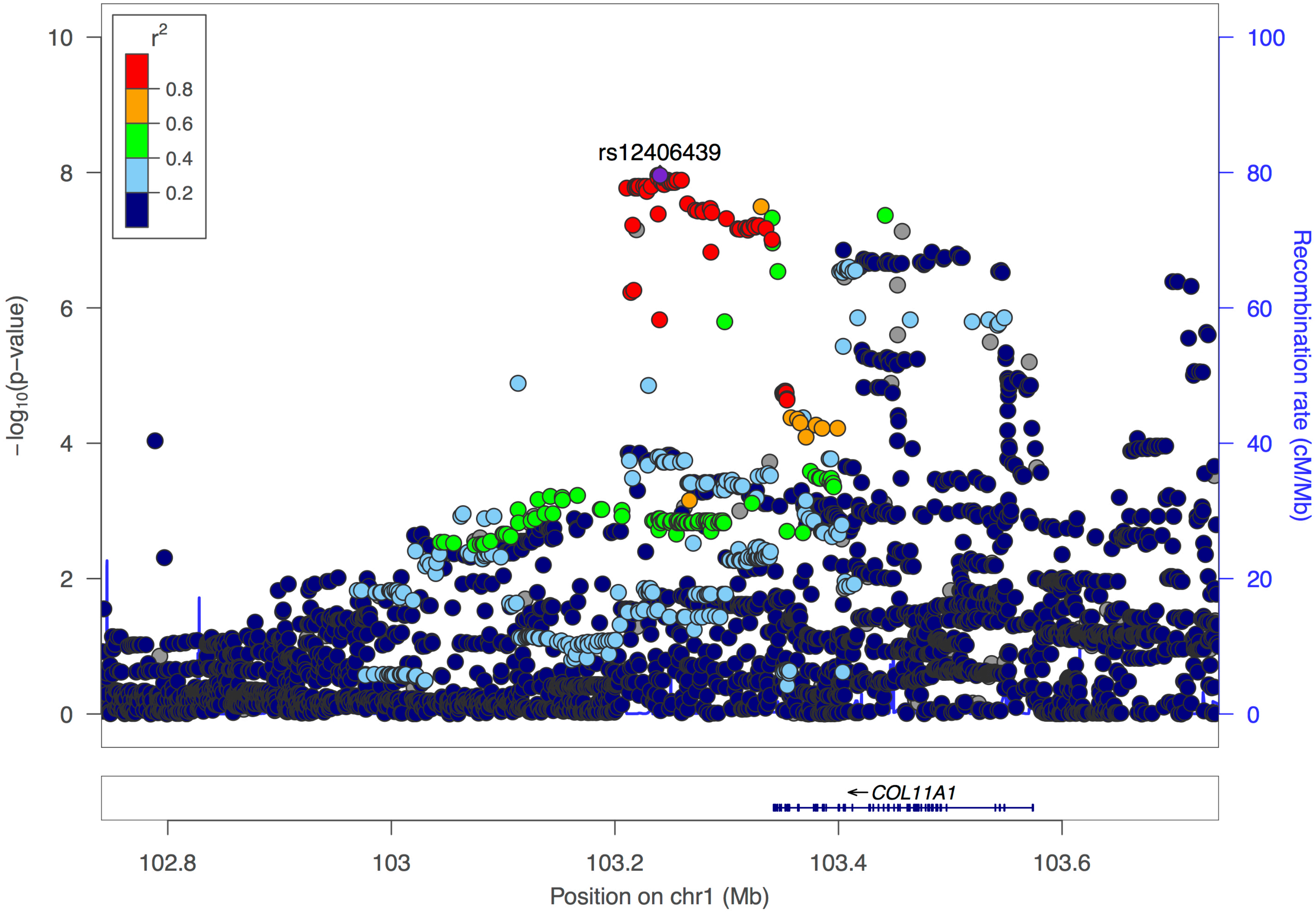

rs12406439
r²
0.8
0.6
0.4
0.2
10
8
6
4
2
0
-log10(p-value)
100
80
60
40
20
0
Recombination rate (cM/Mb)
COL11A1
102.8
103
103.2
103.4
103.6
Position on chr1 (Mb)

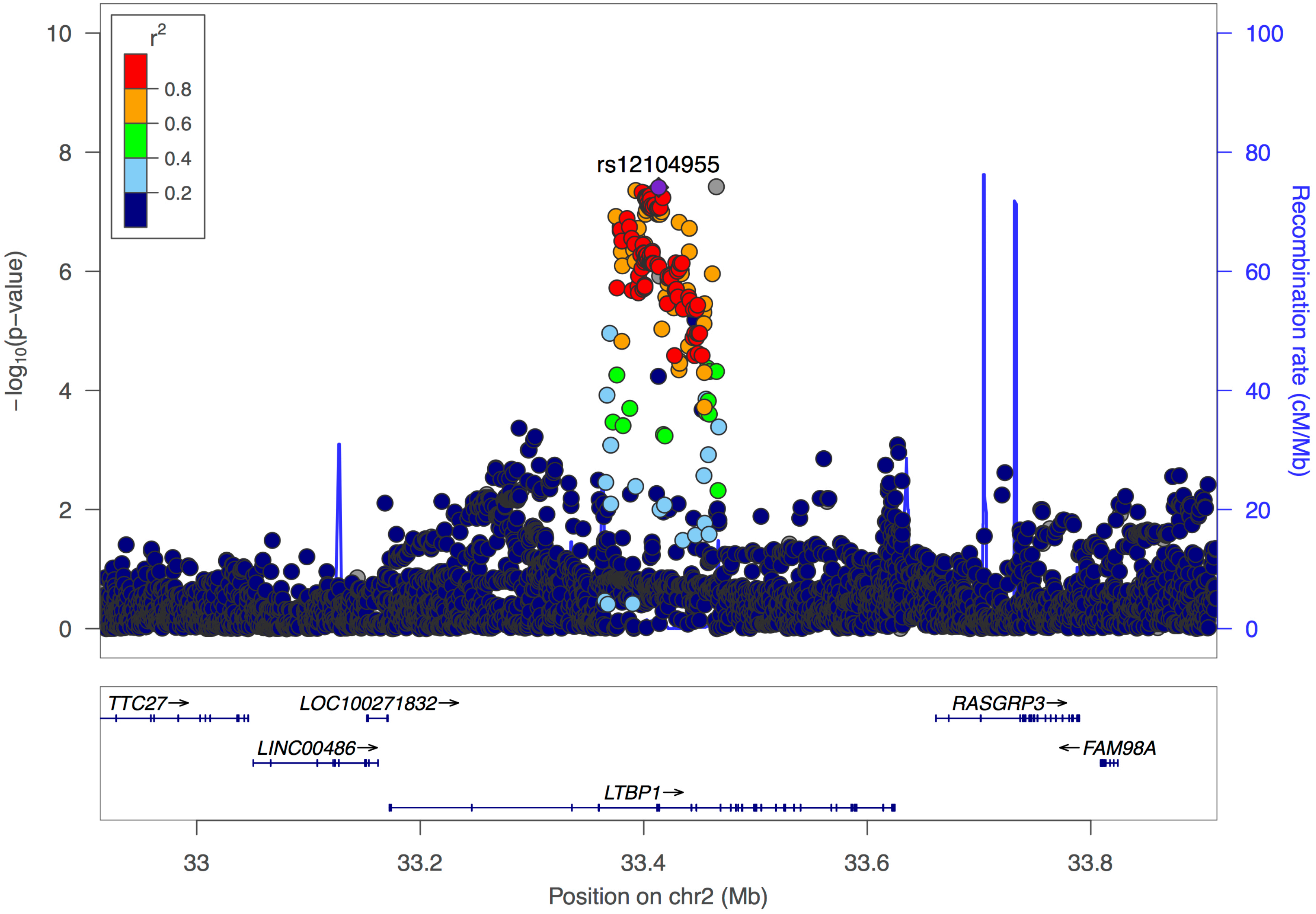

r2
0.8
0.6
0.4
0.2
rs12104955
−log10(p−value)
Recombination rate (cM/Mb)
10
8
6
4
2
0
100
80
60
40
20
0
TTC27→
LOC100271832→
RASGRP3→
LINC00486→
←FAM98A
LTBP1→
33
33.2
33.4
33.6
33.8
Position on chr2 (Mb)

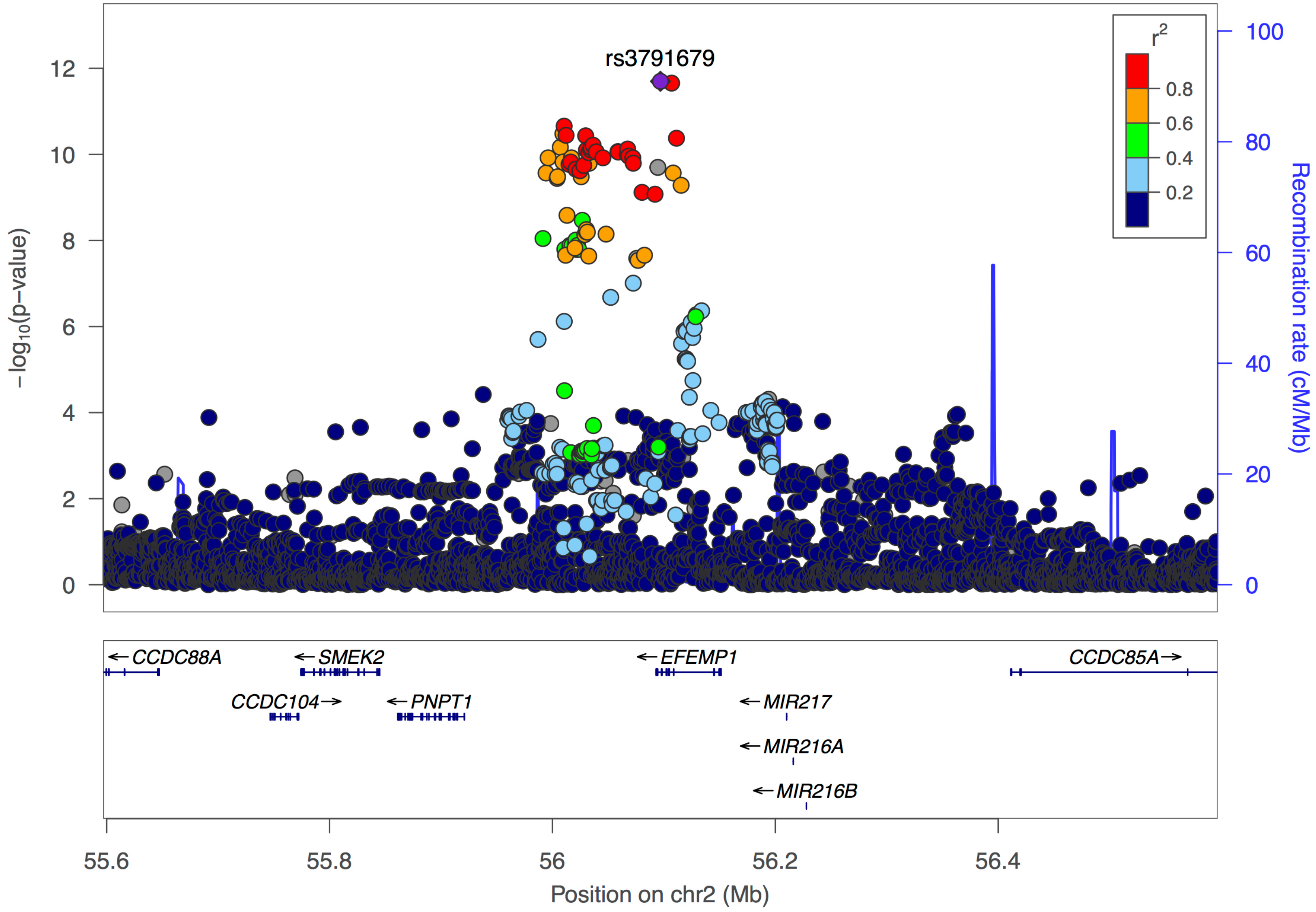

rs3791679
r2
0.8
0.6
0.4
0.2
-log10(p-value)
Recombination rate (cM/Mb)
0
2
4
6
8
10
12
0
20
40
60
80
100
CCDC88A
SMEK2
EFEMP1
CCDC85A
CCDC104
PNPT1
MIR217
MIR216A
MIR216B
55.6
55.8
56
56.2
56.4
Position on chr2 (Mb)

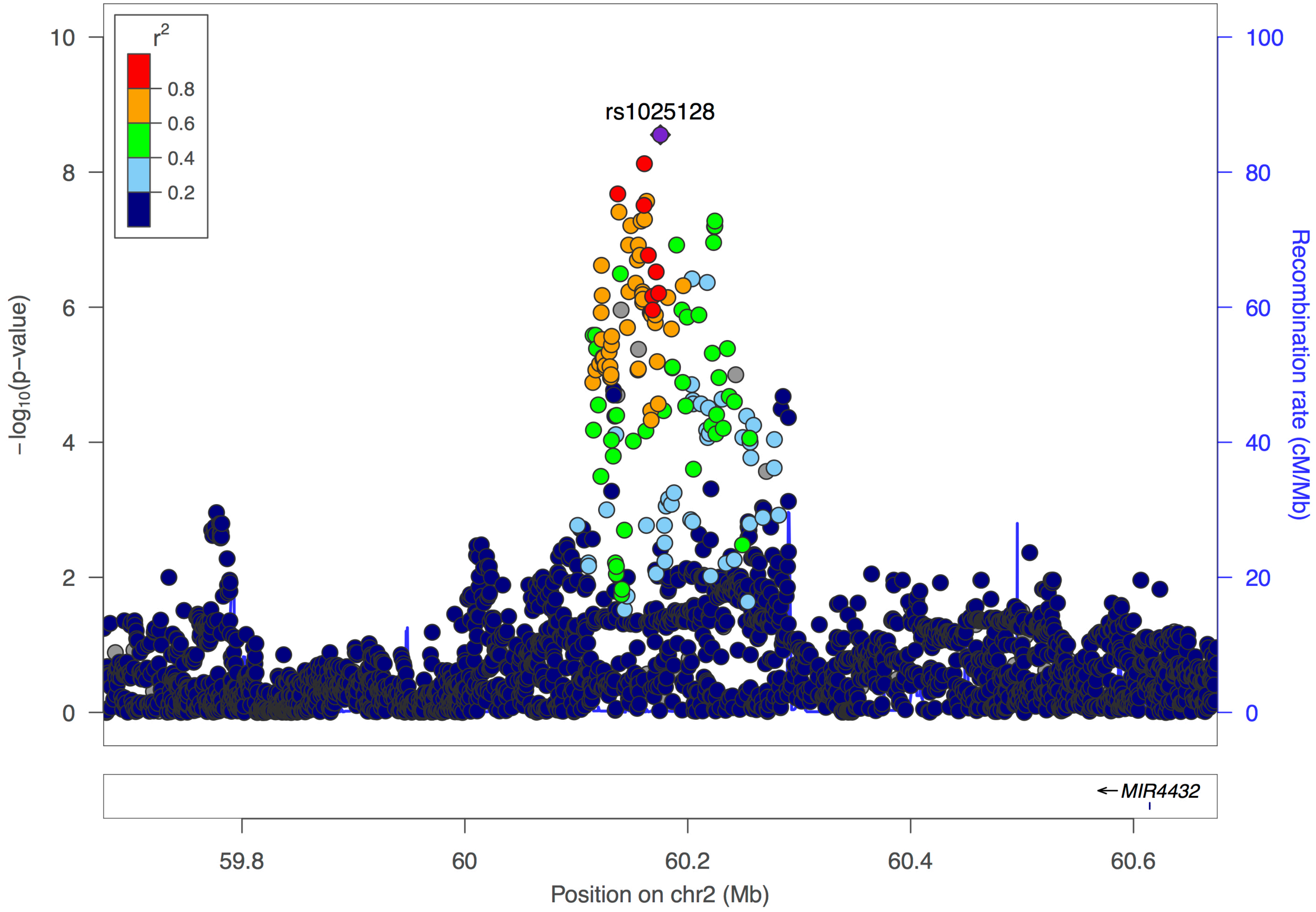

r2
0.8
0.6
0.4
0.2
rs1025128
10
8
6
4
2
0
-log10(p-value)
100
80
60
40
20
0
Recombination rate (cM/Mb)
MIR4432
59.8
60
60.2
60.4
60.6
Position on chr2 (Mb)

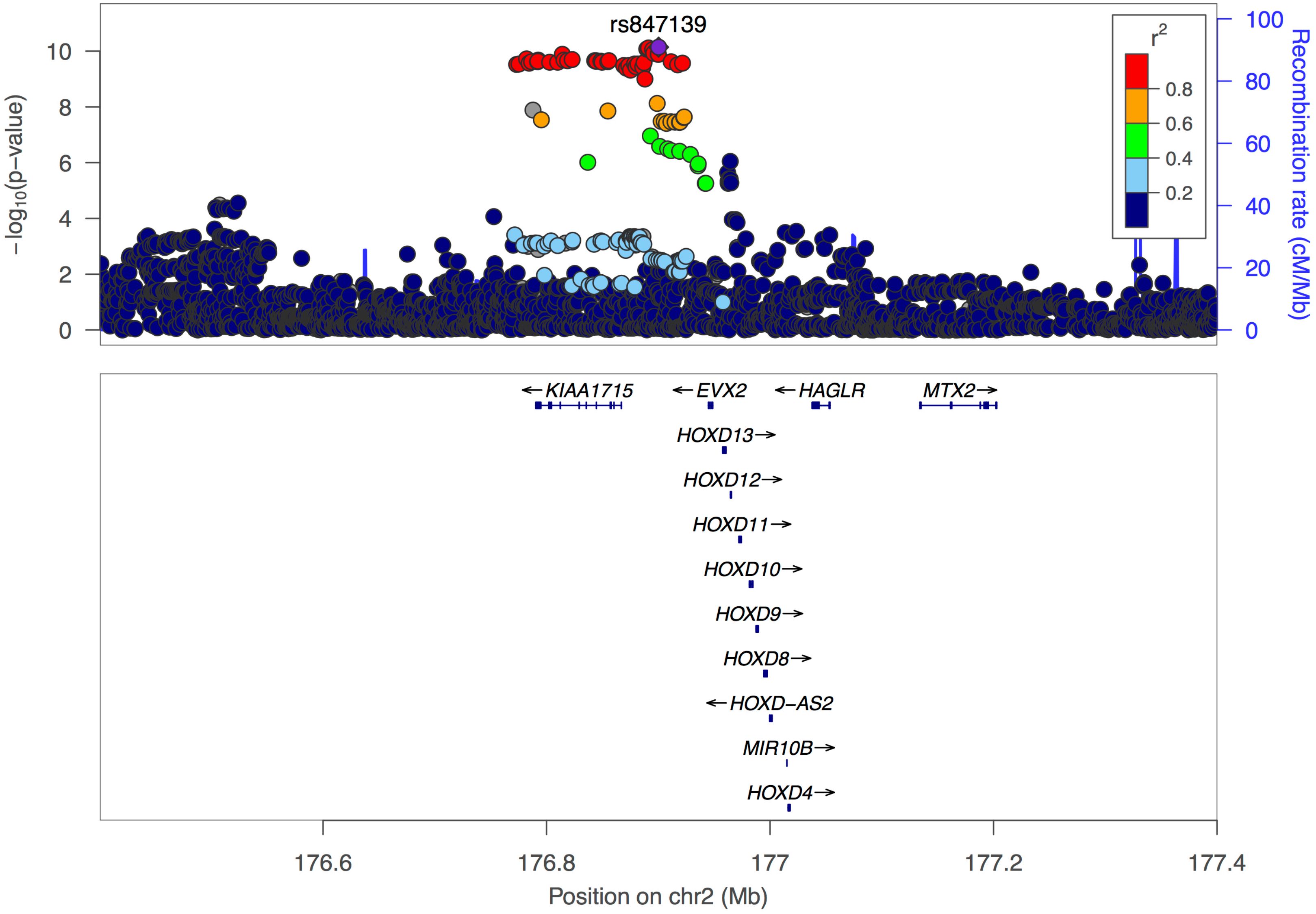
rs847139
r2
0.8
0.6
0.4
0.2
−log10(p−value)
10
8
6
4
2
0
100
80
60
40
20
0
Recombination rate (cM/Mb)
KIAA1715
EVX2
HAGLR
MTX2
HOXD13
HOXD12
HOXD11
HOXD10
HOXD9
HOXD8
HOXD−AS2
MIR10B
HOXD4
176.6
176.8
177
177.2
177.4
Position on chr2 (Mb)

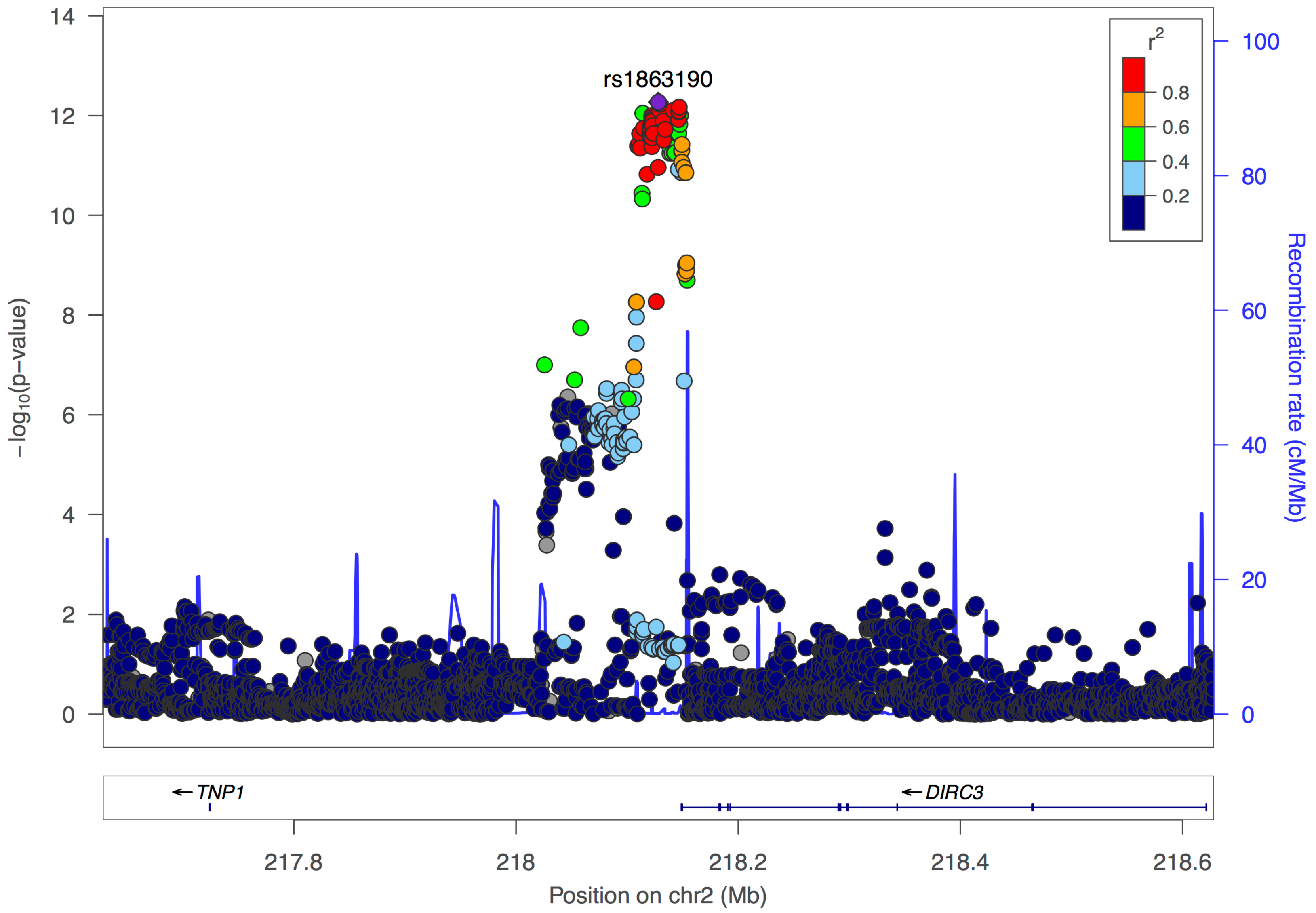
rs1863190
r2
0.8
0.6
0.4
0.2
14
12
10
8
6
4
2
0
-log10(p-value)
100
80
60
40
20
0
Recombination rate (cM/Mb)
← TNP1
← DIRC3
217.8
218
218.2
218.4
218.6
Position on chr2 (Mb)

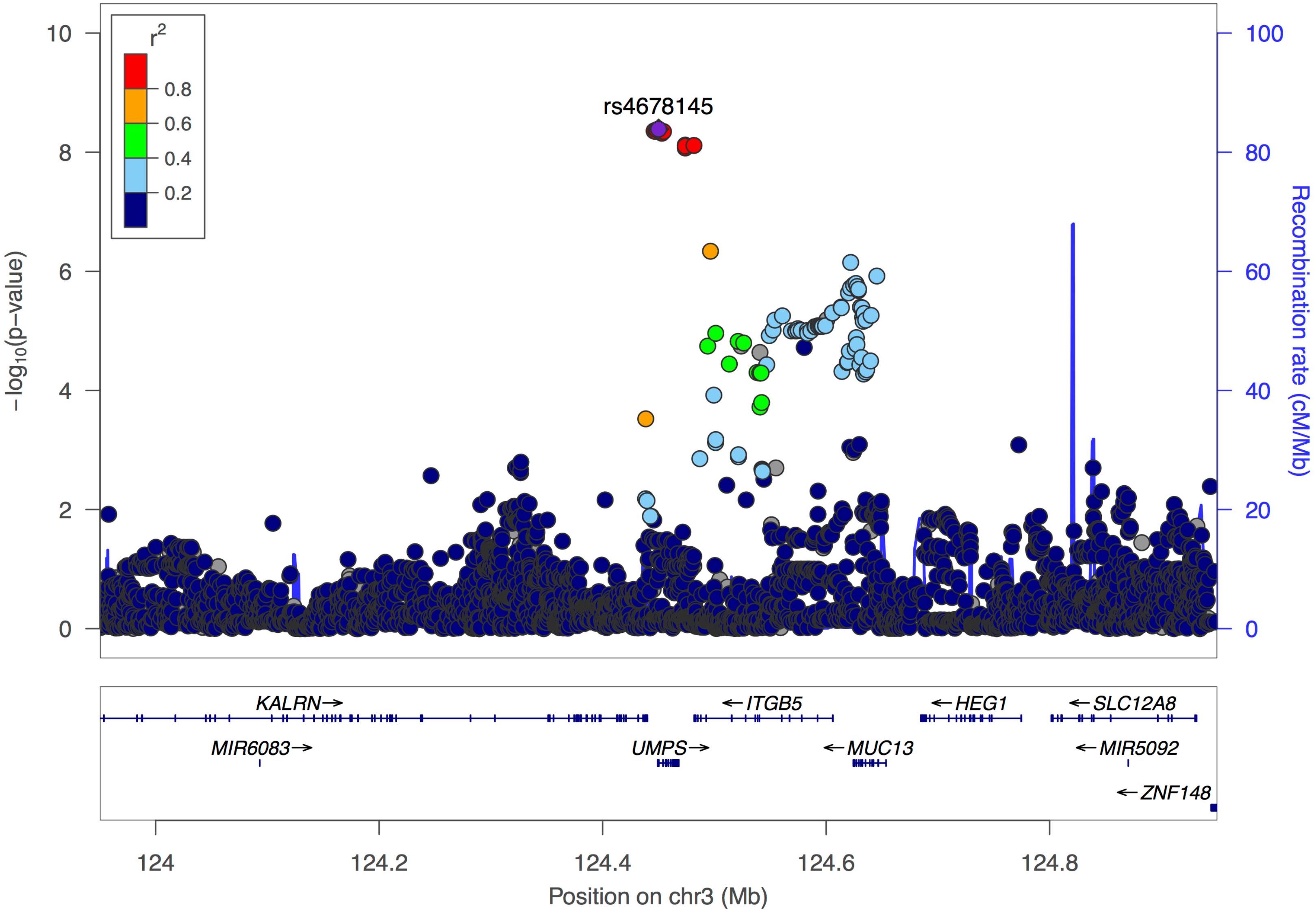

rs4678145
r2
0.8
0.6
0.4
0.2
−log10(p−value)
Recombination rate (cM/Mb)
KALRN→
←ITGB5
←HEG1
←SLC12A8
MIR6083→
UMPS→
←MUC13
←MIR5092
←ZNF148
Position on chr3 (Mb)

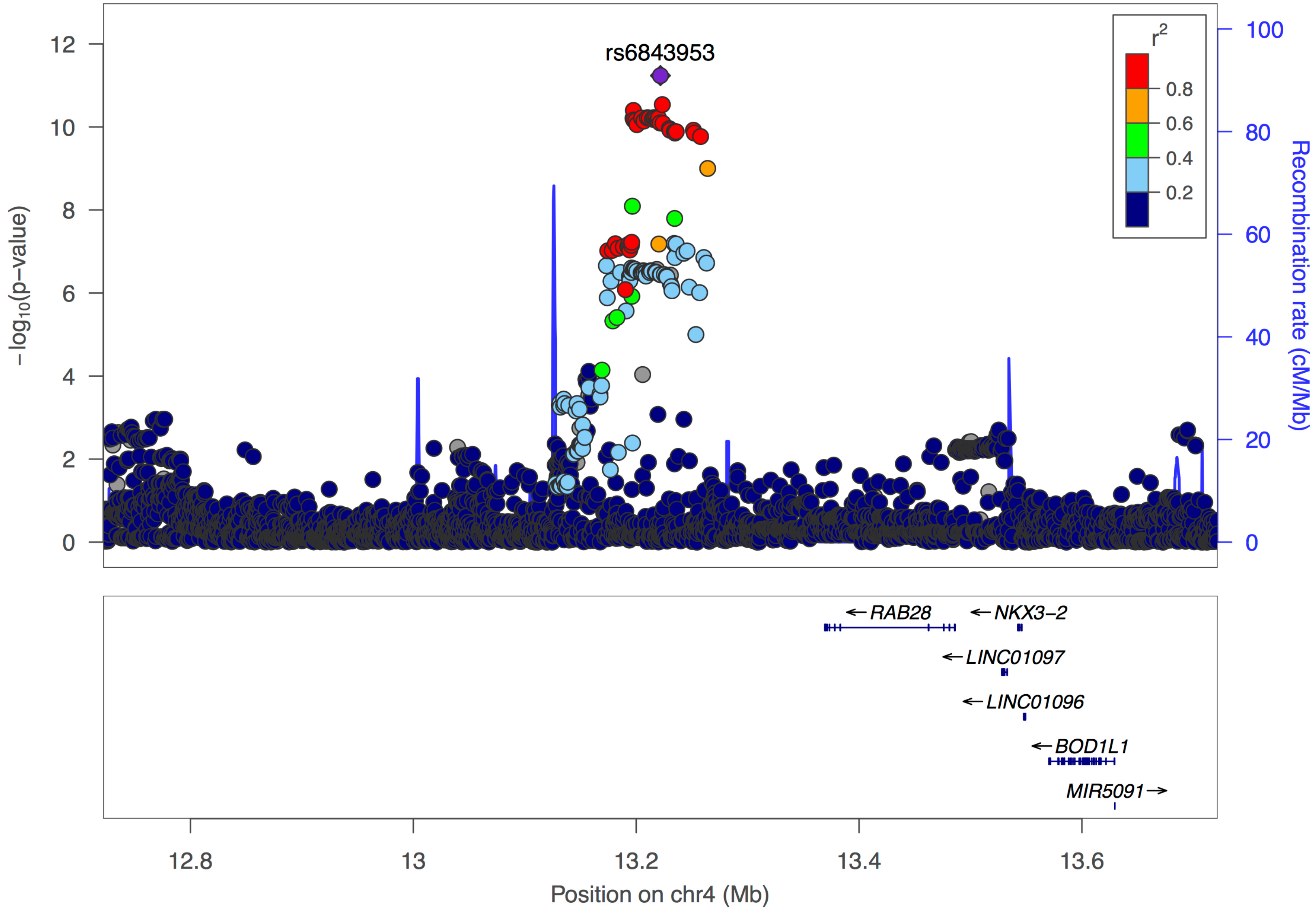
rs6843953
r2
0.8
0.6
0.4
0.2
−log10(p−value)
Recombination rate (cM/Mb)
RAB28
NKX3−2
LINC01097
LINC01096
BOD1L1
MIR5091
12.8
13
13.2
13.4
13.6
Position on chr4 (Mb)

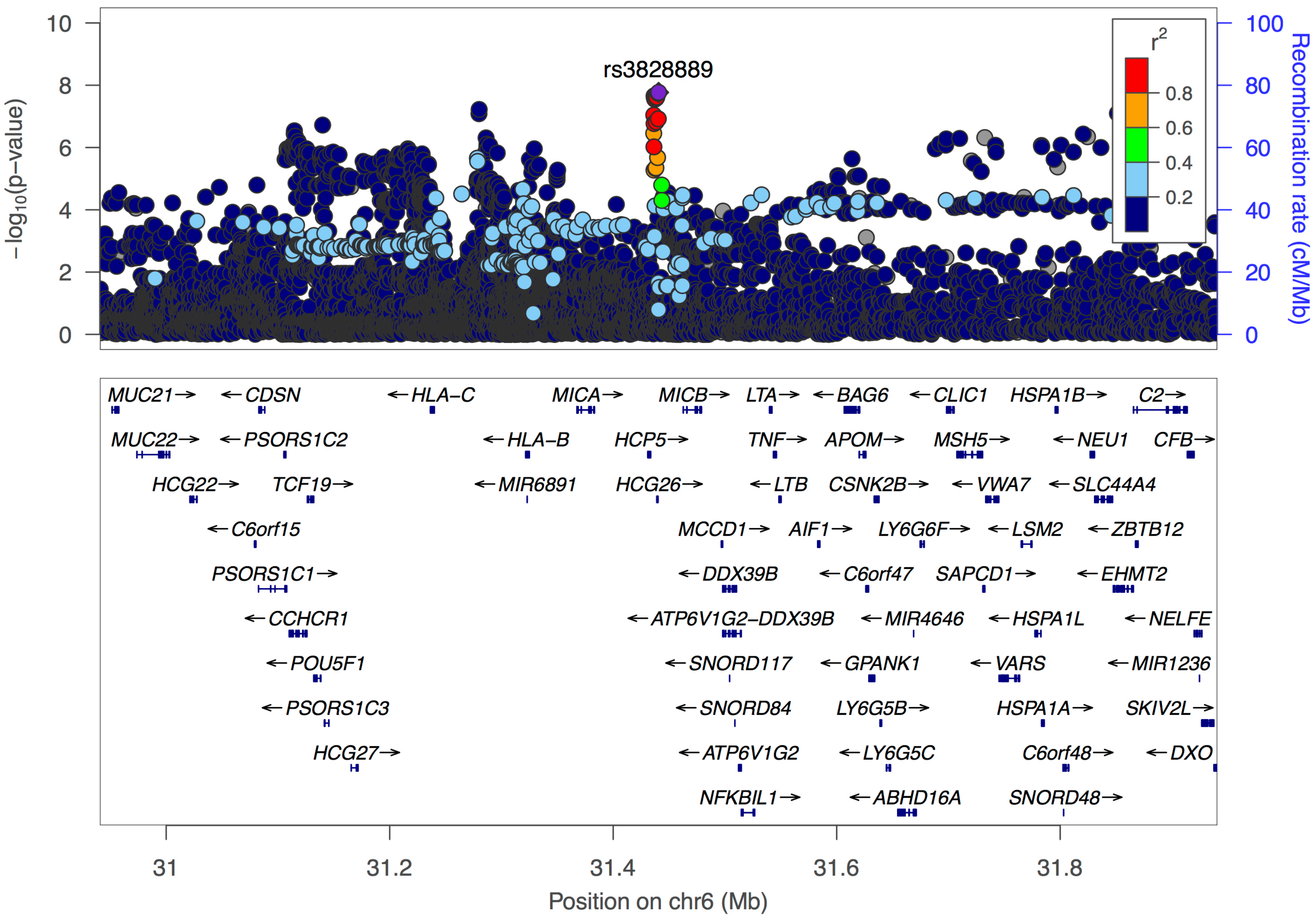

rs3828889
−log10(p−value)
Recombination rate (cM/Mb)
r2
0.8
0.6
0.4
0.2
MUC21→ ←CDSN ←HLA−C MICA→ MICB→ LTA→ ←BAG6 ←CLIC1 HSPA1B→ C2→
MUC22→ ←PSORS1C2 ←HLA−B HCP5→ TNF→ APOM→ MSH5→ ←NEU1 CFB→
HCG22→ TCF19→ ←MIR6891 HCG26→ ←LTB CSNK2B→ ←VWA7 ←SLC44A4
←C6orf15 MCCD1→ AIF1→ LY6G6F→ ←LSM2 ←ZBTB12
PSORS1C1→ ←DDX39B ←C6orf47 SAPCD1→ ←EHMT2
←CCHCR1 ←ATP6V1G2−DDX39B ←MIR4646 ←HSPA1L ←NELFE
←POU5F1 ←SNORD117 ←GPANK1 ←VARS ←MIR1236
←PSORS1C3 ←SNORD84 LY6G5B→ HSPA1A→ SKIV2L→
HCG27→ ←ATP6V1G2 ←LY6G5C C6orf48→ ←DXO
NFKBIL1→ ←ABHD16A SNORD48→
Position on chr6 (Mb)

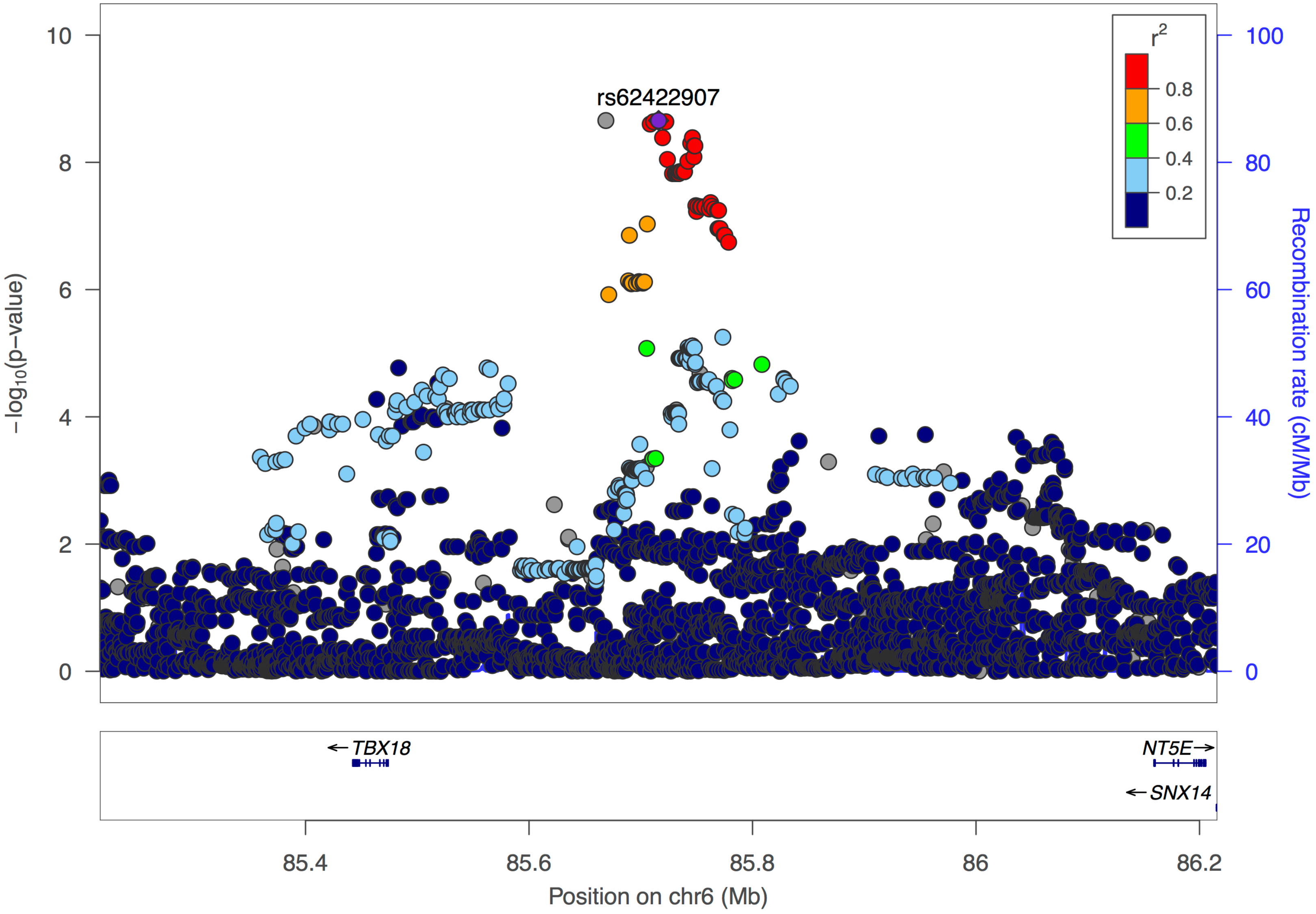

rs62422907
r2
0.8
0.6
0.4
0.2
10
8
6
4
2
0
−log10(p−value)
100
80
60
40
20
0
Recombination rate (cM/Mb)
TBX18
NT5E
SNX14
85.4
85.6
85.8
86
86.2
Position on chr6 (Mb)

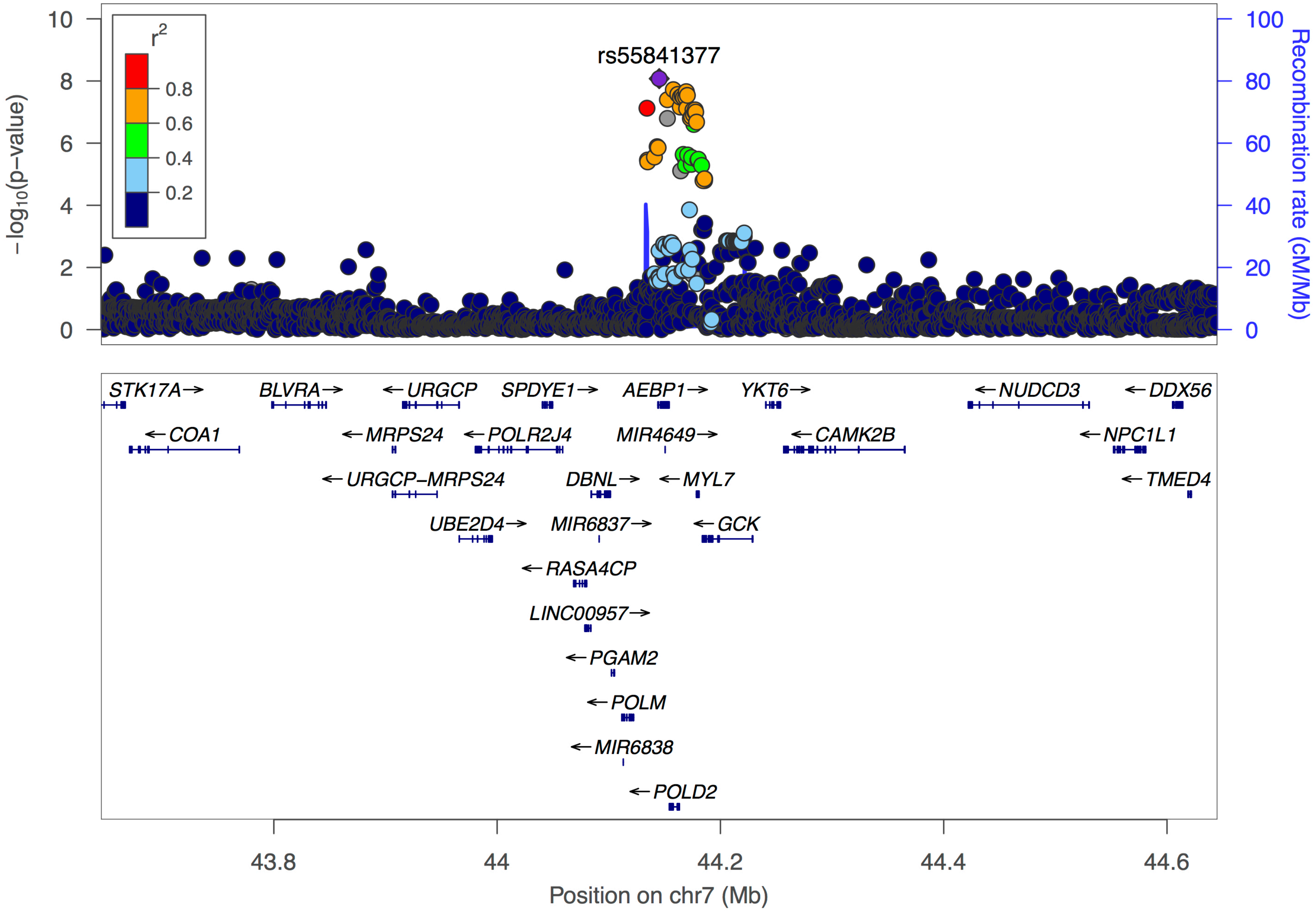

rs55841377
r2
0.8
0.6
0.4
0.2
-log10(p-value)
Recombination rate (cM/Mb)
10
8
6
4
2
0
100
80
60
40
20
0
STK17A→
BLVRA→
←URGCP
SPDYE1→
AEBP1→
YKT6→
←NUDCD3
←DDX56
←COA1
←MRPS24
←POLR2J4
MIR4649→
←CAMK2B
←NPC1L1
←URGCP-MRPS24
DBNL→
←MYL7
←TMED4
UBE2D4→
MIR6837→
←GCK
←RASA4CP
LINC00957→
←PGAM2
←POLM
←MIR6838
←POLD2
43.8
44
44.2
44.4
44.6
Position on chr7 (Mb)

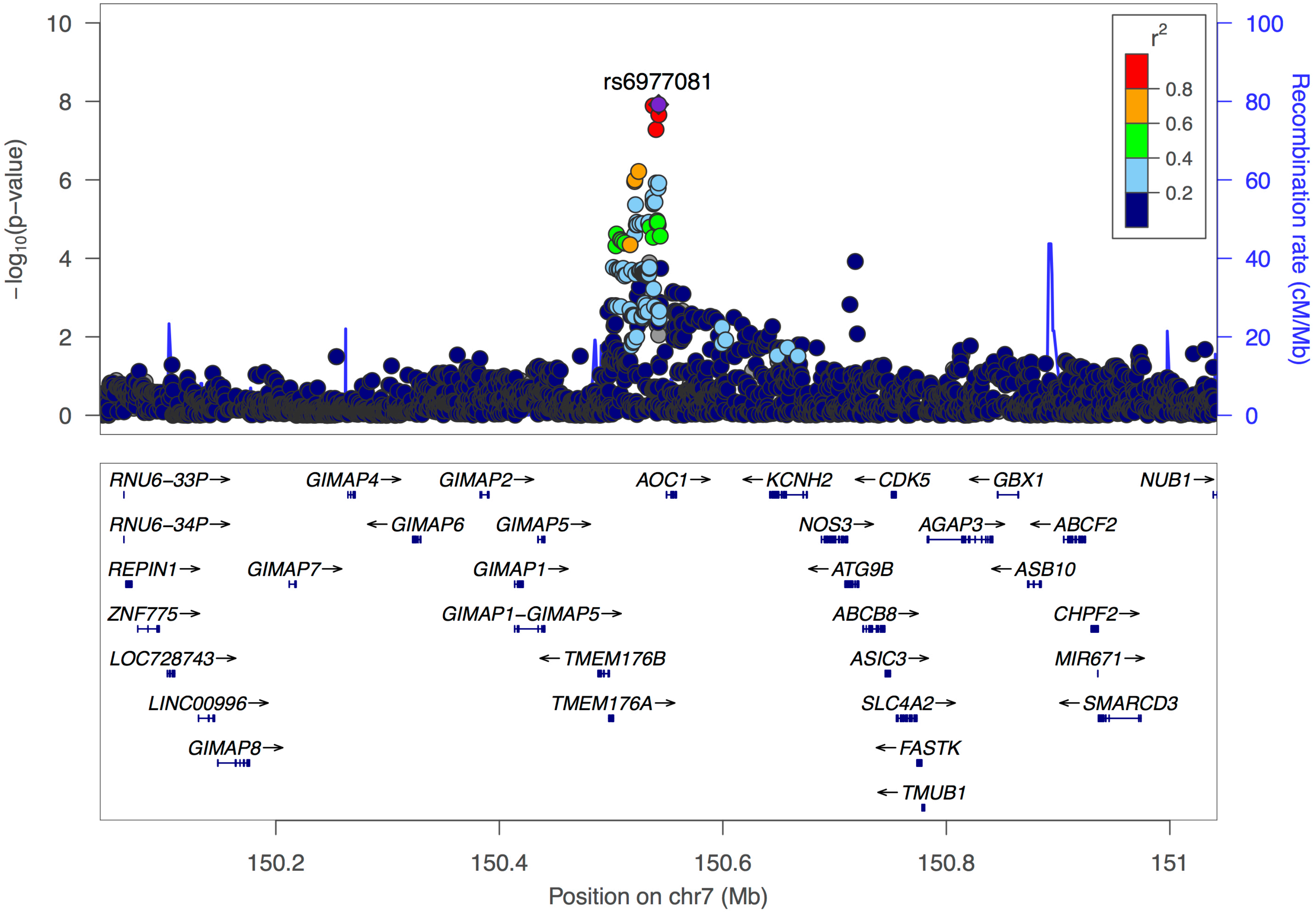
rs6977081
r2
0.8
0.6
0.4
0.2
-log10(p-value)
Recombination rate (cM/Mb)
RNU6-33P→
GIMAP4→
GIMAP2→
AOC1→
←KCNH2
←CDK5
←GBX1
NUB1→
RNU6-34P→
←GIMAP6
GIMAP5→
NOS3→
AGAP3→
←ABCF2
REPIN1→
GIMAP7→
GIMAP1→
←ATG9B
←ASB10
ZNF775→
GIMAP1-GIMAP5→
ABCB8→
CHPF2→
LOC728743→
←TMEM176B
ASIC3→
MIR671→
LINC00996→
TMEM176A→
SLC4A2→
←SMARCD3
GIMAP8→
←FASTK
←TMUB1
150.2
150.4
150.6
150.8
151
Position on chr7 (Mb)

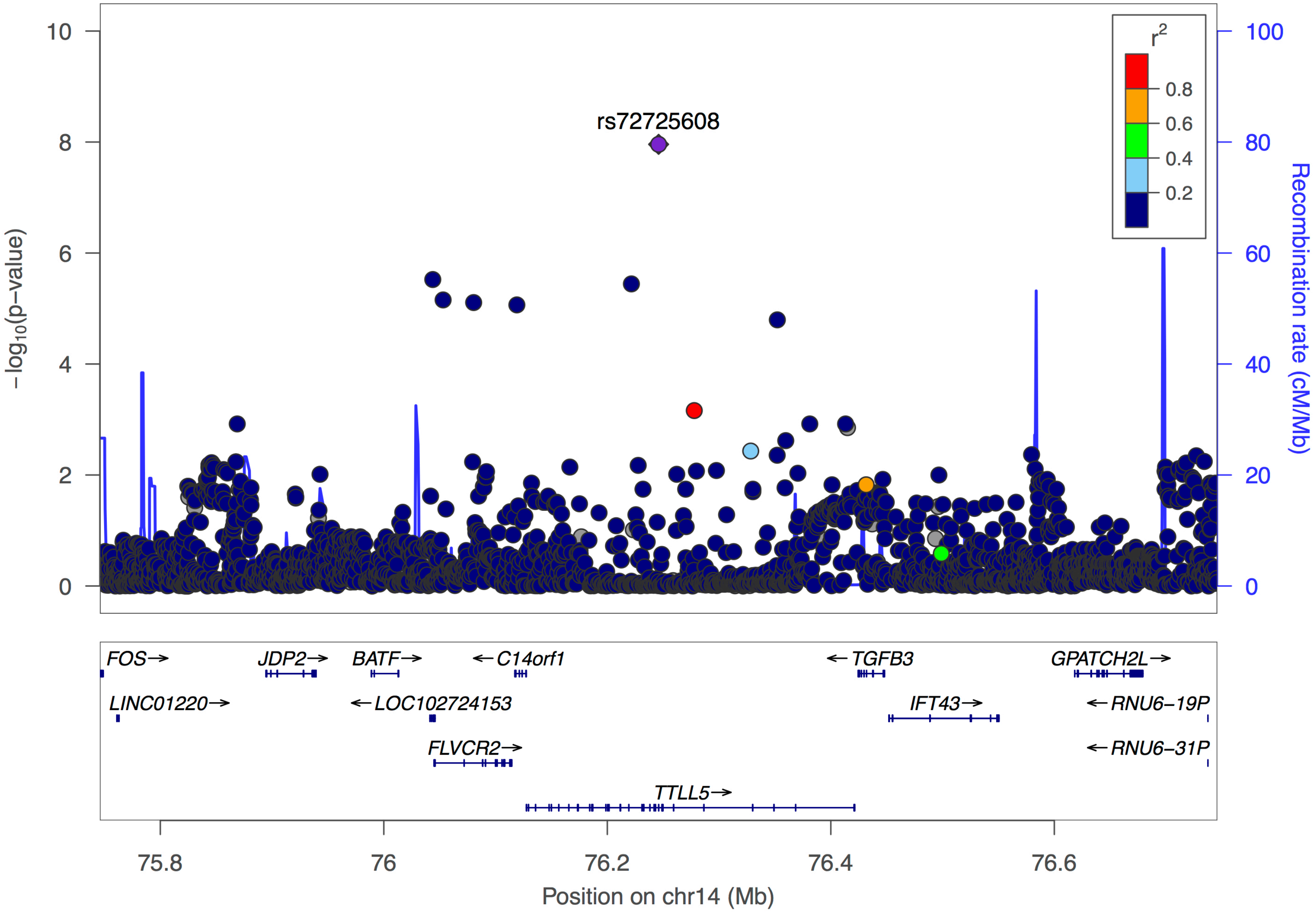
rs72725608
r2
0.8
0.6
0.4
0.2
−log10(p−value)
Recombination rate (cM/Mb)
10
8
6
4
2
0
100
80
60
40
20
0
FOS→
JDP2→
BATF→
←C14orf1
←TGFB3
GPATCH2L→
LINC01220→
←LOC102724153
IFT43→
←RNU6−19P
FLVCR2→
←RNU6−31P
TTLL5→
75.8
76
76.2
76.4
76.6
Position on chr14 (Mb)

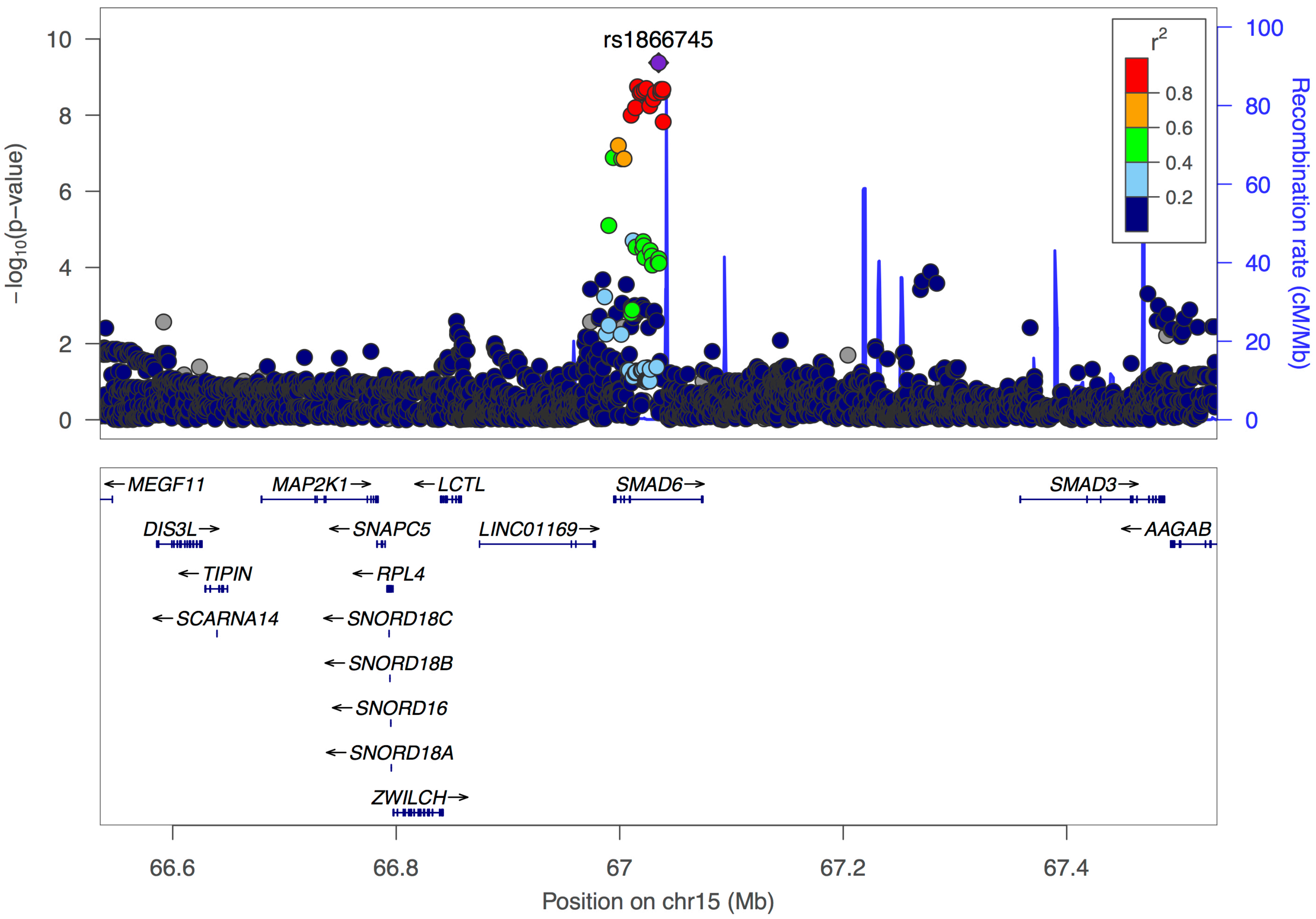
rs1866745
−log10(p−value)
Recombination rate (cM/Mb)
r2
0.8
0.6
0.4
0.2
MEGF11
MAP2K1
LCTL
SMAD6
SMAD3
DIS3L
SNAPC5
LINC01169
AAGAB
TIPIN
RPL4
SCARNA14
SNORD18C
SNORD18B
SNORD16
SNORD18A
ZWILCH
66.6
66.8
67
67.2
67.4
Position on chr15 (Mb)

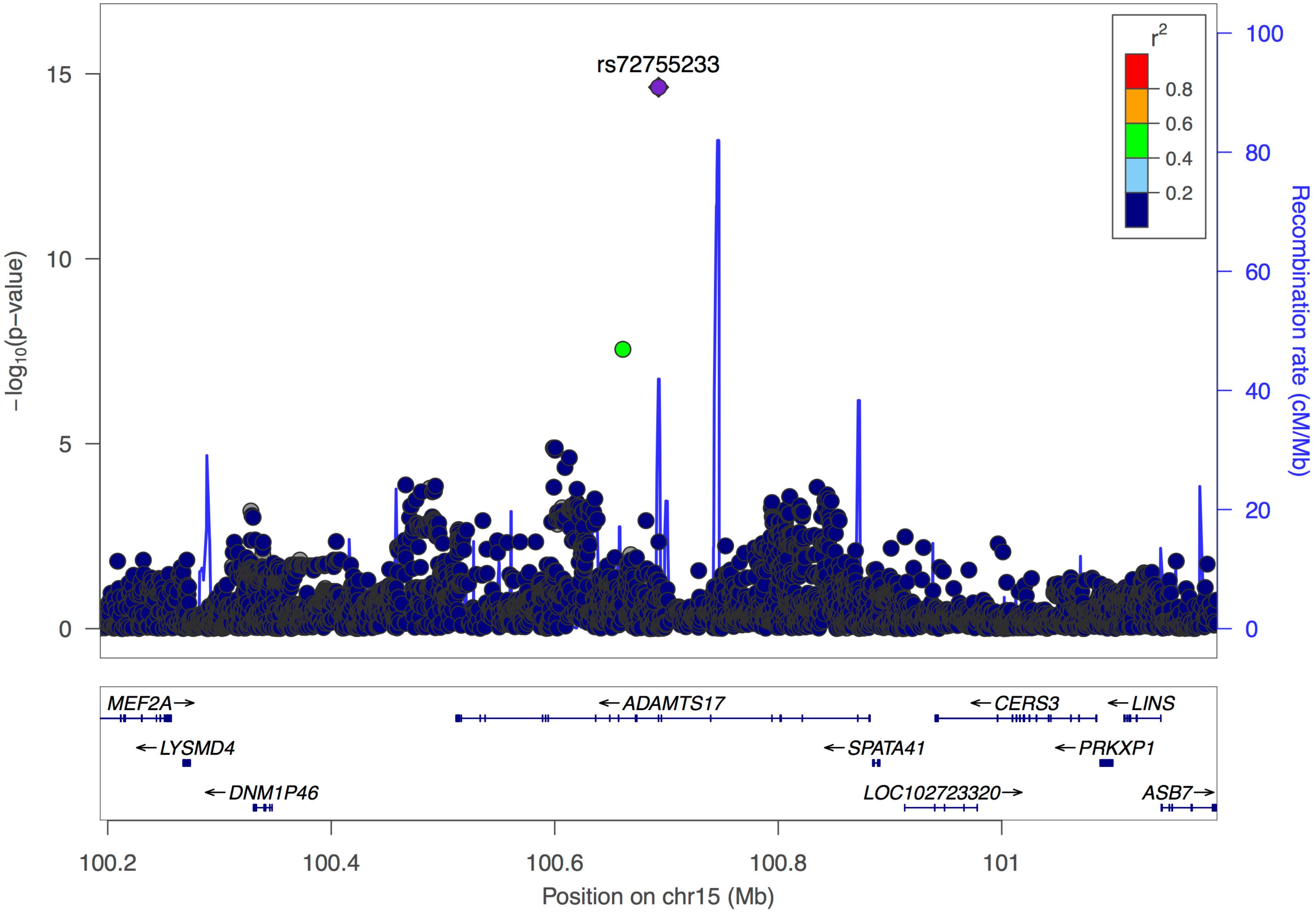

rs72755233
r2
0.8
0.6
0.4
0.2
−log10(p−value)
15
10
5
0
100
80
60
40
20
0
Recombination rate (cM/Mb)
MEF2A→
←ADAMTS17
←CERS3
←LINS
←LYSMD4
←SPATA41
←PRKXP1
←DNM1P46
LOC102723320→
ASB7→
100.2
100.4
100.6
100.8
101
Position on chr15 (Mb)

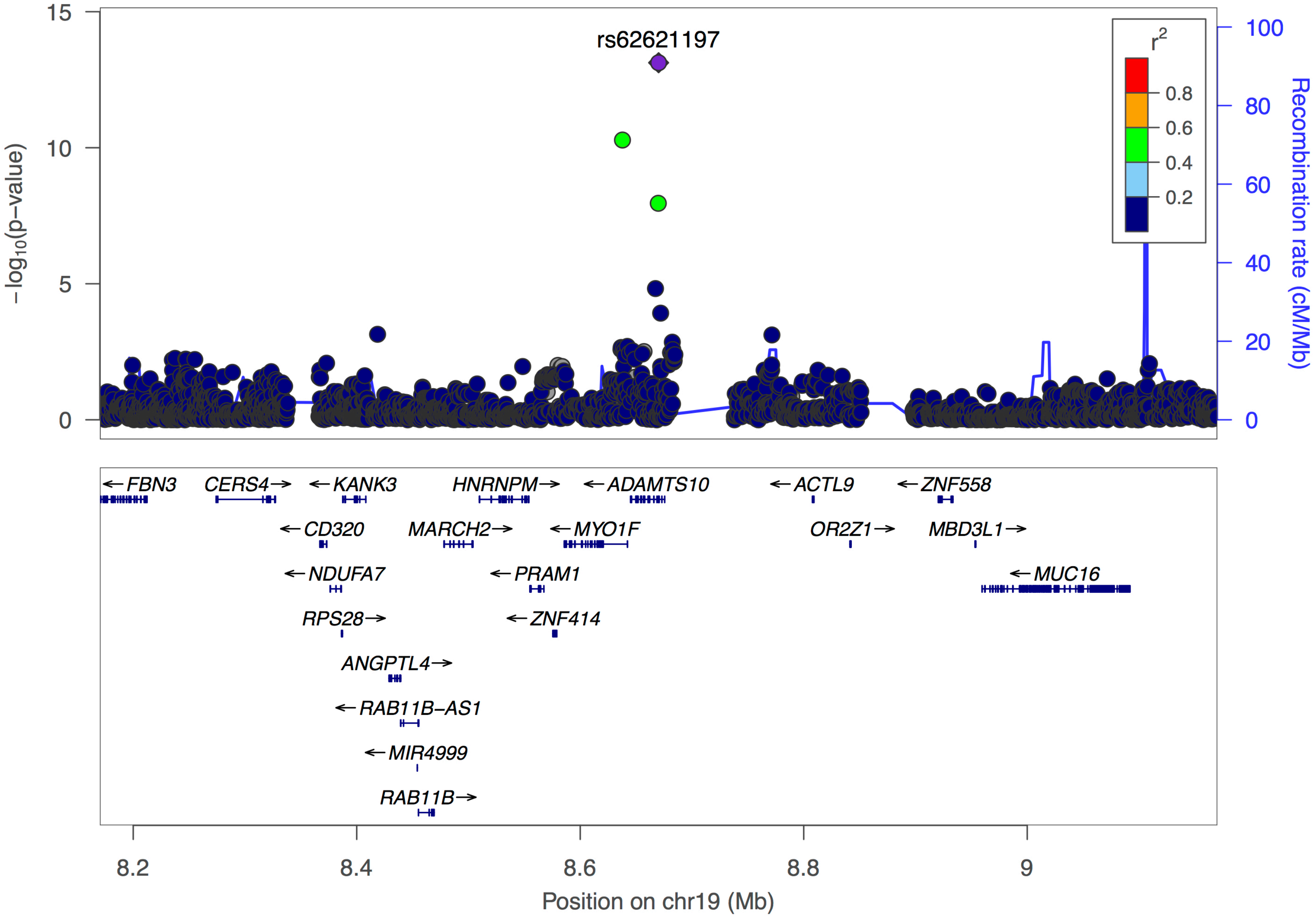

rs62621197
15
10
5
0
−log10(p−value)
r2
0.8
0.6
0.4
0.2
100
80
60
40
20
0
Recombination rate (cM/Mb)
←FBN3
CERS4→
←KANK3
HNRNPM→
←ADAMTS10
←ACTL9
←ZNF558
←CD320
MARCH2→
←MYO1F
OR2Z1→
MBD3L1→
←NDUFA7
←PRAM1
←MUC16
RPS28→
←ZNF414
ANGPTL4→
←RAB11B−AS1
←MIR4999
RAB11B→
8.2
8.4
8.6
8.8
9
Position on chr19 (Mb)

Supplementary Figure 7. Regional Locus Zoom plots of all GWAS-associated loci. LocusZoom plots of the 16 top-associated SNPs, ordered by chromosome number and genomic position. SNP position is shown on the x-axis, and strength of association on the y-axis. The linkage disequilibrium (LD) relationship between the lead SNP and the surrounding SNPs is indicated by the colour. In the lower panel of each figure, genes within 500kb of the index SNP are shown. The position on each chromosome is shown in relation to Human Genome build hg19.

Supplementary Discussion

Replication in Estonian Cohort

i) Background

We performed the first ever GWAS of Carpal Tunnel Syndrome (CTS), which is also the first ever GWAS in an entrapment neuropathy. Using a powerful resource such as UK Biobank allowed us to perform an association study with over 12,000 robustly phenotyped cases. Finding a suitably sized replication cohort proved challenging in a disease such as CTS, which has hitherto been relatively under-investigated from a genetic perspective. However, the Estonian Genome Center at the University of Tartu (EGCUT) identified approximately 4,000 CTS cases in their Biobank, and a replication study was therefore attempted.

ii) Methods

Cohort Description, Genotyping and Imputation

The Estonian Biobank is a population-based biobank of the Estonian Genome Center at the University of Tartu (EGCUT). All 51,886 participants have signed a broad informed consent form, which allows periodical linking to national registries, electronic health record databases and hospital information systems[2]. High-coverage whole genome sequencing data is available for the 2,535 individuals, selected randomly by county of birth. Biobank participants have been analysed using Illumina genotyping arrays: 1) Global Screening Array (GSA, N=33,277), 2) HumanCoreExome (CE, N=7,832), 3) HumanOmniExpress (OMNI, N=8,137), and 4) 370K (N=2,640). If samples were genotyped with different arrays, the following order of beadchips was preferred: GSA, CE, OMNI, 370K. The final set of genotyped data contained 48,163 unique individuals. Individuals with missing phenotype data were excluded.

The genotype calling for the microarrays was performed using Illumina's GenomeStudio v2010.3 software. The genotype calls for rare variants on the GSA array were corrected using the zCall software (version May 8th, 2012). After variant calling, the data were filtered using PLINK[3] v.1.90 by sample (call rate >95%, no sex mismatches between phenotype and genotype data, heterozygosity < mean ±3 SE) and by marker (HWE $P > 1\times10^{-6}$, call rate > 95%, and for the GSA array additionally by Illumina GenomeStudio GenTrain score >0.6, Cluster Separation Score >0.4). Before imputation, variants with MAF < 1% and C/G or T/A polymorphisms as well as indels were removed, as these genotype calls do not allow precise phasing and imputation.

Pre-phasing of genotyped data was performed using Eagle[4] v2.3 with default parameters, but --Kpbwt=20000 was specified to increase accuracy for all four arrays separately with Eagle and imputed with BEAGLE[5] v4.1, using a population-specific imputation reference panel[6]. All four imputed arrays were merged into a single dataset with BCFtools (https://samtools.github.io/bcftools/bcftools.html). Imputation information measures (INFO-value) were added using plugin 'impute-info' separately for both IRPs, monomorphic and multi-allelic imputed variants were excluded.

A description of the phenotyping methodology in EGCUT is described in detail elsewhere[2]. Case ascertainment performed using a mixture of self-reported and physician-confirmed CTS diagnoses identified a total of 4,438 CTS cases and 45,958 controls.

Statistical Analyses

The association analysis was performed using an additive model in SNPTEST[7], using year of birth, sex and the first 10 principal components as covariates. We extracted the relevant summary statistics for the 16 SNPs that were genome-wide significant in the discovery GWAS, and calculated the weighted genetic risk score for CTS in both cases and controls in the EGCUT cohort using the summary statistics from the UK Biobank discovery GWAS (our method has been described in the main manuscript). We also performed LD score regression[8] to estimate the degree of genetic correlation between the UK Biobank and EGCUT summary statistics.

iii) Results

Demographics:

The demographic characteristics of the two cohorts are shown side-by-side, separated by sex. The overall female preponderance was 54.1% in UK Biobank and 65.9% in EGCUT.

		UKB Cases	UKB Controls	UKB All	EGCUT Cases	EGCUT Controls	EGCUT All
**Males**	N	3,738	180,761	184,499	783	16,390	17,173
	Prevalence	0.02			0.046		
	Age (s.d.)	69.0 (7.4)	67.0 (7.4)	67.1 (8.1)	49.8 (14.9)	41.9 (18.0)	42.3 (17.9)
	Height (s.d.)	173.8 (6.7)	175.9 (6.7)	175.9 (6.8)	176.6 (6.8)	178.8 (7.3)	178.7 (7.3)
	Weight (s.d.)	90.35 (15.9)	86.1 (14.2)	86.2 (14.3)	88.4 (16.3)	84.1 (15.6)	84.2 (15.7)
**Females**	N	8,574	208,583	217,157	3,655	29,568	33,223
	Prevalence	0.039			0.11		
	Age (s.d.)	68.3 (7.1)	66.6 (8.0)	66.6 (7.9)	49.5 (13.2)	43.9 (17.6)	44.5 (17.2)
	Height (s.d.)	160.7 (6.2)	162.7 (6.2)	162.6 (6.2)	163.4 (6.1)	165.0 (6.5)	164.8 (6.5)
	Weight (s.d.)	75.3 (15.9)	71.4 (13.8)	71.5 (13.9)	76.1 (16.8)	70.3 (14.8)	71.0 (15.2)

The graph below demonstrates the age distribution of the EGCUT cohort:

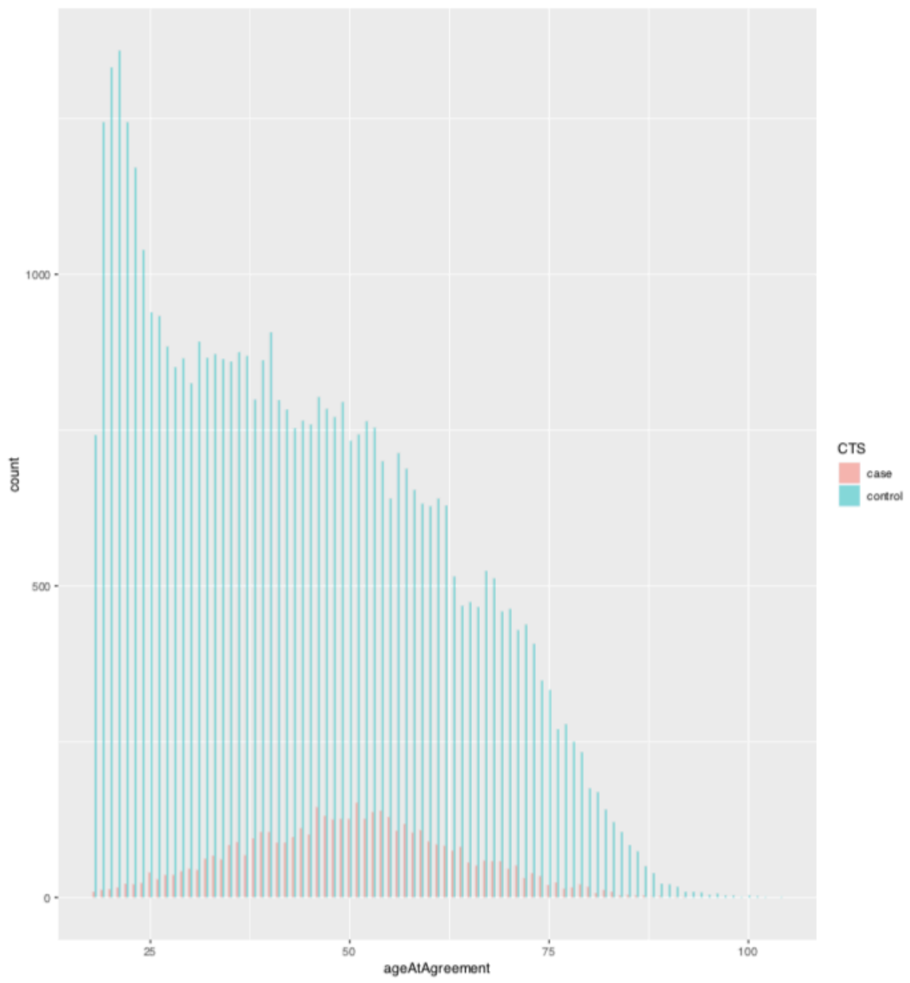

count
1000
500
0
25
50
75
100
ageAtAgreement
CTS
case
control

Summary statistics:

The summary statistics for the 16 genome-wide significant discovery SNPs in the EGCUT cohort are shown below, alongside the summary statistics from the discovery GWAS in the UK Biobank cohort. P-values are unadjusted.

	UK BIOBANK					EGCUT					
rsID	Effect Allele	EAF Cases	EAF Controls	OR (95% CI)	P-value	Effect Allele	EAF Cases	EAF Controls	(95% CI)	P-value	Directionally concordant?
rs12406439	T	0.605	0.587	1.08 (1.05-1.11)	1.10×10^{-8}	T	0.627	0.617	1.04 (0.99-1.09)	0.050	Yes
rs12104955	C	0.517	0.499	1.07 (1.05-1.10)	3.90×10^{-8}	C	0.455	0.447	1.03 (0.99-1.08)	0.116	Yes
rs3791679	G	0.244	0.225	1.11 (1.08-1.15)	2.00×10^{-12}	G	0.276	0.271	1.02 (0.98-1.08)	0.327	Yes
rs1025128	C	0.582	0.563	1.08 (1.05-1.11)	2.80×10^{-9}	C	0.676	0.668	1.03 (0.99-1.09)	0.105	Yes
rs847139	C	0.805	0.787	1.11 (1.07-1.14)	7.20×10^{-11}	C	0.820	0.815	1.03 (0.97-1.09)	0.169	Yes
rs1863190	T	0.780	0.760	1.12 (1.08-1.15)	5.40×10^{-13}	T	0.708	0.701	1.03 (0.98-1.08)	0.264	Yes
rs4678145	G	0.880	0.868	1.12 (1.08-1.16)	4.10×10^{-9}	G	0.914	0.905	1.10 (1.02-1.20)	0.014	Yes
rs6843953	T	0.154	0.138	1.14 (1.10-1.18)	5.80×10^{-12}	T	0.096	0.0924	1.04 (0.97-1.13)	0.338	Yes
rs3828889	C	0.748	0.732	1.09 (1.06-1.12)	1.70×10^{-8}	C	0.692	0.694	0.99 (0.95-1.04)	0.757	No
rs62422907	G	0.899	0.887	1.13 (1.09-1.18)	2.20×10^{-9}	G	0.911	0.916	0.94 (0.86-1.01)	0.088	No
rs55841377	C	0.789	0.773	1.09 (1.06-1.13)	8.40×10^{-9}	C	0.841	0.838	1.02 (0.95-1.08)	0.500	Yes
rs6977081	G	0.685	0.668	1.08 (1.05-1.11)	1.20×10^{-8}	G	0.610	0.603	1.03 (0.98-1.08)	0.239	Yes
rs72725608	C	0.051	0.044	1.20 (1.13-1.27)	1.10×10^{-8}	C	0.0335	0.031	1.09 (0.96-1.24)	0.165	Yes
rs1866745	A	0.369	0.35	1.09 (1.06-1.12)	4.20×10^{-10}	A	0.324	0.325	0.997 (0.95-1.24)	0.848	No
rs72755233	A	0.128	0.112	1.18 (1.13-1.22)	2.30×10^{-15}	A	0.145	0.139	1.05 (0.99-1.12)	0.086	Yes
rs62621197	T	0.045	0.036	1.31 (1.22-1.40)	7.50×10^{-14}	T	0.049	0.042	1.18 (1.06-1.31)	0.002	Yes

Weighted Genetic Risk Score:

	GRS in CTS cases	GRS in controls
UK Biobank	1.620	1.566
EGCUT	1.519	1.142

L.D. Score Regression

The genetic correlation (r_g) between UK Biobank and EGCUT GWAS of CTS was 0.9044 (SE 0.0031), Z score = 5.76, p = 8.19×10^{-9}.

iv) Discussion

In this replication study, 13 out of 16 loci discovered in the UK Biobank discovery GWAS were directionally concordant in the EGCUT replication cohort, but only one SNP (rs62621197) achieved significance at a Bonferroni-corrected p-value threshold of 0.0031 (0.05/16). However, there is significant genetic correlation between the two cohorts, as evidenced by the correlation coefficient of 0.90 on LD score regression; furthermore, the separation of weighted genetic risk scores between cases and controls in EGCUT showed a similar pattern to the UK Biobank cohort.

There are significant limitations to this replication study that need to be highlighted. Firstly, there is a significant discrepancy between the mean ages of the CTS cases in the two cohorts (69 in UK Biobank vs 50 in EGCUT). The graph showing the age distribution of the EGCUT cases and controls demonstrates a near-normal distribution of CTS cases around the mean age of 50. Given that idiopathic CTS is very rare below the age of 40, it is particularly striking that the overall prevalence of CTS in the EGCUT cohort is substantially higher than in UK Biobank (8.5% vs 3.1%), even though the ECCUT cases are nearly 20 years younger than the UK Biobank cases. UK Biobank participants were recruited at ages 40-69 (the peak age for CTS incidence), and the prevalence was still under 5%. This leads us to conclude that the EGCUT cohort likely includes a substantial proportion of pregnancy-related CTS – a common and usually transient phenomenon due to fluid retention in pregnancy, which is quite distinct in terms of pathophysiology from idiopathic CTS. Further evidence for this is the discrepancy in the proportion of female CTS cases: 70% in UK Biobank vs 83% in EGCUT.

In terms of case ascertainment, in UK Biobank, we performed a validation study of CTS diagnoses by scrutinising hundreds of medical records of putative CTS cases, and determined that the positive predictive value of a CTS 'case' being a true case is 94%[9]. In addition, 94% of our CTS cases have at least one operation code, suggesting that our cohort are, on the whole, on the phenotypically more severe end of the spectrum. Finally, 80% of our CTS cases have at least one ICD-10 or OPCS code for CTS, meaning that our reliance on self-reported questionnaire diagnoses is very low.

However, the principal reason for the relative lack of replication of our SNPs in the EGCUT cohort is likely lack of statistical power. Despite having >4,000 cases, this still only represents approximately one-third of the cases included in the UK Biobank discovery cohort. With controls included, the EGCUT cohort is only 12% of the size of the UK Biobank cohort. Based on the number of Estonian cases and controls and the allelic odds ratios from our GWAS, we calculated the power to detect an association at the Bonferroni-corrected p-value threshold of p=0.0031, assuming a population disease prevalence of 5%. While we acknowledge the significant problems with *post hoc* calculations of statistical power[10], it is apparent from the table below that we were significantly underpowered at the majority of loci; only at 4/16 loci do we have > 80% power to detect an association at this threshold, and that is assuming that the majority of the 4,087 cases are true cases of idiopathic CTS (as opposed to pregnancy-related CTS).

SNP	Power (4087 cases)
rs12406439	0.61
rs12104955	0.51
rs3791679	0.88
rs1025128	0.63
rs847139	0.56
rs1863190	0.92
rs4678145	0.39
rs6843953	0.67
rs3828889	0.68
rs62422907	0.5
rs55841377	0.39
rs6977081	0.63
rs72725608	0.45
rs1866745	0.73
rs72755233	0.99
rs62621197	0.97

In summary, there are significant limitations in this replication attempt pertaining to case ascertainment, discrepancies in age and sex distribution and disease prevalence, and lack of power. Nevertheless, there is clearly strong evidence for shared genetic architecture for CTS between the two cohorts, as evidenced by the genetic risk scores, genetic correlation and directional concordance of the effect at the majority of loci. While EGCUT is a robust cohort for performing population-based studies, we feel that a formal replication study and meta-analysis needs to be conducted in a replication cohort of larger or comparable size to UK Biobank, where we can also be confident that we are comparing the same phenotype (i.e. idiopathic, rather than pregnancy-related CTS) between the discovery and replication samples.

Supplementary References

1. Weiss T, Taschner-Mandl S, Bileck A, Slany A, Kromp F, Rifatbegovic F, Frech C, Windhager R, Kitzinger H, Tzou CH, Ambros PF, Gerner C, A. I. Proteomics and transcriptomics of peripheral nerve tissue and cells unravel new aspects of the human Schwann cell repair phenotype. *Glia* **64,** 2133–2153 (2016).
2. Leitsalu, L. *et al.* Cohort profile: Estonian biobank of the Estonian genome center, university of Tartu. *Int. J. Epidemiol.* **44,** 1137–1147 (2015).
3. Purcell, S. *et al.* PLINK: A Tool Set for Whole-Genome Association and Population-Based Linkage Analyses. *Am. J. Hum. Genet.* **81,** 559–575 (2007).
4. Loh, P. R. *et al.* Reference-based phasing using the Haplotype Reference Consortium panel. *Nat. Genet.* **48,** 1443–1448 (2016).
5. Browning, B. L. & Browning, S. R. Genotype Imputation with Millions of Reference Samples. *Am. J. Hum. Genet.* **98,** 116–126 (2016).
6. Mitt, M. *et al.* Improved imputation accuracy of rare and low-frequency variants using population-specific high-coverage WGS-based imputation reference panel. *Eur. J. Hum. Genet.* **25,** 869–876 (2017).
7. Marchini, J., Howie, B., Myers, S., McVean, G. & Donnelly, P. A new multipoint method for genome-wide association studies by imputation of genotypes. *Nat. Genet.* **39,** 906–913 (2007).
8. Bulik-Sullivan, B. *et al.* An atlas of genetic correlations across human diseases and traits. *Nat. Genet.* **47,** 1236–1241 (2015).
9. Lane, J. *et al.* Can we use routinely collected hospital and GP data for epidemiological study of common hand conditions? A UK Biobank based validation project. Preprint at https://www.biorxiv.org/content/10.1101/274167v1 (2018).
10. Greenland, S. Nonsignificance Plus High Power Does Not Imply Support for the Null Over the Alternative. *Ann. Epidemiol.* **22,** 364–368 (2012).
