# Supplementary material for: A genome-wide association analysis identifies 16 novel susceptibility loci for carpal tunnel syndrome
Source: Nat Commun. 2019 Mar 4;10:1030. doi: 10.1038/s41467-019-08993-6 (PMC6399342; doi:10.1038/s41467-019-08993-6)
Supplement: Supplementary file 3 — Description of Additional Supplementary Files [file 41467_2019_8993_MOESM3_ESM.docx]

**Description of Additional Supplementary Files**

**Supplementary Data 1.** 422 SNPs significantly associated (p<5**×**10^-8^) in the CTS GWAS.

**Supplementary Data 2.** Genome-wide significant SNPs with likely functional consequences.

**Supplementary Data 3.** Full GWAS summary statistics.
